# Application Progress of Organoids in Colorectal Cancer

**DOI:** 10.3389/fcell.2022.815067

**Published:** 2022-02-22

**Authors:** Lianxiang Luo, Yucui Ma, Yilin Zheng, Jiating Su, Guoxin Huang

**Affiliations:** ^1^ The Marine Biomedical Research Institute, Guangdong Medical University, Zhanjiang, China; ^2^ The Marine Biomedical Research Institute of Guangdong Zhanjiang, Zhanjiang, China; ^3^ Southern Marine Science and Engineering Guangdong Laboratory (Zhanjiang), Zhanjiang, China; ^4^ Clinical Research Center, Shantou Central Hospital, Shantou, China; ^5^ The First Clinical College, Guangdong Medical University, Zhanjiang, China

**Keywords:** organoids, colorectal cancer, living biobank, drug screening, personalized medicine, drug toxicity, regeneration medicine

## Abstract

Currently, colorectal cancer is still the third leading cause of cancer-related mortality, and the incidence is rising. It is a long time since the researchers used cancer cell lines and animals as the study subject. However, these models possess various limitations to reflect the cancer progression in the human body. Organoids have more clinical significance than cell lines, and they also bridge the gap between animal models and humans. Patient-derived organoids are three-dimensional cultures that simulate the tumor characteristics *in vivo* and recapitulate tumor cell heterogeneity. Therefore, the emergence of colorectal cancer organoids provides an unprecedented opportunity for colorectal cancer research. It retains the molecular and cellular composition of the original tumor and has a high degree of homology and complexity with patient tissues. Patient-derived colorectal cancer organoids, as personalized tumor organoids, can more accurately simulate colorectal cancer patients’ occurrence, development, metastasis, and predict drug response in colorectal cancer patients. Colorectal cancer organoids show great potential for application, especially preclinical drug screening and prediction of patient response to selected treatment options. Here, we reviewed the application of colorectal cancer organoids in disease model construction, basic biological research, organoid biobank construction, drug screening and personalized medicine, drug development, drug toxicity and safety, and regenerative medicine. In addition, we also displayed the current limitations and challenges of organoids and discussed the future development direction of organoids in combination with other technologies. Finally, we summarized and analyzed the current clinical trial research of organoids, especially the clinical trials of colorectal cancer organoids. We hoped to lay a solid foundation for organoids used in colorectal cancer research.

## Introduction

Colorectal cancer (CRC) ranked the third one in tumor incidence of all malignant tumors and the second one in cancer-related mortality worldwide (Keum and Giovannucci, 2019). According to the global cancer statistics in 2018, there were nearly 1.8 million new cases and more than 800,000 deaths, accounting for 10.2% of all incident cancer cases and 9.2% of the total number of cancer deaths, respectively (Bray et al., 2018). The incidence of CRC in developed countries has declined significantly. However, it is rising rapidly in developing countries (Siegel et al., 2019). Data from the Global Cancer Observatory (https://gco.iarc.fr/today) showed that there were approximately 520,000 new cases of CRC in China and approximately 240,000 deaths. The number of new cases and deaths was close to 28% of the global data in the same period, which seriously endangered people’s health.

CRC is a complex and multifactorial disease that involves genetic and environmental risk factors (Yang et al., 2019), including positive family history, hereditary CRC syndrome, inflammatory bowel disease, a high-fat diet, deficiency in dietary fiber, obesity. Moreover, smoking, lacking physical exercise, and age are also closely related to CRC (Nguyen et al., 2018). CRC is generally an asymptomatic disease before it progresses to advanced stages. In clinics, many CRC patients were diagnosed at a more advanced stage (Roney et al., 2021). Early endoscope screening is essential for the diagnosis of CRC which takes at least 5–10 years for adenomas to develop into CRC. CRC Statistics in the United States indicated the rapid decline in the incidence of CRC of people over 55 years because of the widespread use of early endoscope screening (Siegel et al., 2017). Colonoscopy is considered the most sensitive and specific method for lesion detection and monitoring among the various screening methods used for CRC. It can detect and remove most precancerous adenomas of the early stage. However, colonoscopy is an invasive examination that causes discomfort and potential complications for the patients, which restricts the widespread application in physical examinations (Alves Martins et al., 2019). The clinical data showed the cure rate approaching 90% after surgery when CRC is diagnosed in the early stage. However, it was only 10% of the 5-years survival rate of patients with metastatic CRC that cancer cells spread to distant lymph nodes or other organs (Relier et al., 2016). The survival rate of patients with advanced CRC has been substantially improved due to the use of chemotherapeutics, the introduction of targeted therapies for specific tumor characteristics, and the combination of multidisciplinary methods. The median overall survival reaches 30 months in clinical trials. Despite advances in therapeutic strategies, the mortality rate of CRC remains at a high level (Kuipers et al., 2015). Relevant reports have proposed that CRC be classified according to its molecular subtypes. The unique treatment strategy was applied for different types of CRC patients that facilitated the individualized treatment of CRC (Guinney et al., 2015). Currently, individual treatment of cancer patients is generally based on gene sequencing in the clinics, but only 7% of the beneficiary population received personalized medicine based on next-generation sequencing (NGS) (Meric-Bernstam et al., 2015). However, the development of drugs resistance in patients receiving anticancer therapies is a continuing problem and the main obstacle to effective treatment (Jung et al., 2021). The current research and preclinical development of antitumor drugs are based on traditional tumor biology research models, including conventional two-dimensional cell lines (2D) and patient-derived tumor xenografts (PDXs). However, cell lines do not have the integrative microenvironment of living tissues and cannot retain tumor genetic information and heterogeneity in the process of passage. Because murine gene characteristics and growth environment are different from tumor patients, PDXs models undergo mouse-specific tumor evolution (Zhou et al., 2017; Mittal et al., 2019). Moreover, this model has a low success rate, high cost, and is time consuming, so that it cannot better satisfy demand (Ben-David et al., 2017). So, it is urgent to develop a new model for tumor research.

The first organoid was successfully established by Hans Clevers in 2009 (Sato et al., 2009). Since then, organoid has increasingly gained in popularity. In recent years, organoid has become a research hotspot, showing significant promising in the biological analysis of the tumor. Organoids, a kind of *in vitro* culture system, contains self-renewing stem cells that differentiate into various organ-specific cell types and tissues, a three-dimensional (3D) structure that assumes a similar organization and functionality as an organ. It overcomes many limitations of traditional models. These organoids are closer to cell composition, behavior, and physiology of native tissues and have more stable genomic structures. At present, cancer organoids are derived from tumor tissues of mice or humans. In the appropriate culture conditions, organoids formed a 3D structure that is similar to mice or human tumors, and mostly maintained tumor tissue structure, the gene lineage, and tumor heterogeneity. Tumor organoids can be used to study the mechanism of occurrence and development of disease and tissue transplant in regenerative medicine, which is also an effective screening platform for high-throughput drug screening. Currently, drug discovery *in vitro* is a relatively successful application to identify personalized drug combinations and more efficient treatment plans for individual patients (Fatehullah et al., 2016; Xu et al., 2018a; Xu et al., 2018b; Li and Izpisua Belmonte, 2019). In this article, we aimed to summarize the application of organoids in CRC by showing basic research, preclinical data and systematically reviewing early clinical evidence available to highlight the most promising avenues for clinical leveraging of organoids in CRC.

## CRC Organoids

Organoids are 3D constructs that grow *in vitro* and are self-renewing and self-organizing (Sato et al., 2009; Lancaster et al., 2013). Organoids can be derived from embryonic stem cells (ESCs), induced pluripotent stem cells (iPSCs), adult stem cells (ASCs), and even tumor cells (Sato et al., 2009; Lancaster et al., 2013; Lancaster and Huch, 2019). Its functions and structures are highly similar to the organs (Tuveson and Clevers, 2019). Since Clevers et al. (Sato et al., 2009) successfully cultured small intestine organs from mice in 2009, multiple organoids have been successfully produced and reported, including the intestine (Sato et al., 2009; Fujii et al., 2018), heart (Hoang et al., 2018), liver (Huch et al., 2015; Tüysüz et al., 2017), pancreas (Broutier et al., 2016), kidney (Takasato et al., 2015; Low et al., 2019), prostate (Gao et al., 2014; Karthaus et al., 2014), lung (Rock et al., 2009; Wong et al., 2012; Nikolić et al., 2017; Sachs et al., 2019), retina (Xie et al., 2020), and brain (Lancaster et al., 2013). Moreover, a variety of cancer organoids have been successfully established, which includes breast cancer (Sachs et al., 2018; Dekkers et al., 2020), lung cancers (Kim et al., 2019; Shi R. et al., 2020), CRC (Karthaus et al., 2014; Matano et al., 2015), and so on. At present, cancer cell lines are still one of the widely applied models for large-scale drug screening. However, this model also has shortcomings that lack spatial structure and overall microenvironment *in vivo* and cannot simulate tumor heterogeneity (Xuefeng et al., 2020). There were significant differences between 2D cultured cell lines and 3D cultured cell models in drug sensitivity (Imamura et al., 2015). The patient-derived tumor xenograft (PDX) model can simulate the situation *in vivo* to a certain extent, which can better maintain tumor heterogeneity and genetic complexity *in vivo* (Jiang S et al., 2020). However, this model still faces many problems, including a low successful rate of transplantation tumors, large tumor sample requirement, and a long experimental period (Lau et al., 2020). The PDOs models cover the shortage of existing models and offer unique advantages. It can proliferate indefinitely *in vitro* and maintain the heterogeneity of tumors. Therefore, organoids can be widely used in tumor studies of tumor occurrence, development, and drug response.

In the field of CRC, organoids were first isolated and established in mice by Sato (Sato et al., 2009), and 2 years later, he developed a protocol that allowed a robust and long-term culture of primary human epithelial cells isolated from the colon (Sato et al., 2011). Subsequent studies on CRC organoids mostly adopted Sato’s protocols or were slightly modified based on these protocols. Tissue is mechanically dissociated and then digested into single cells. In the isolation procedure, Rho kinase inhibitor (Y-27632) is included in the medium to avoid anoikis, which seems to determine the survival rate. Cells are plated into matrigel and cultured in a basal culture medium. Here we list different culture mediums for culturing mouse and human organoids (Table 1). Obviously, gastrin, A83-01, and SB202190 are added to the human organoid medium compared with the mouse organoid medium. The addition of gastrin can extend the survival time of organoids. A83-01, an inhibitor of Alk4/5/7, can significantly improve the plating efficiency. SB202190 can inhibit p38, inhibiting goblet cell differentiation and increasing intestinal epithelial proliferation.

**TABLE 1:** Summary of the different methods related to CRC organoids of mouse and human.

References	Medium	Tissues
Sato Sato et al. (2009)	AdvDMEM/F12, 10–50 ng/ml EGF,500 ng/ml R-spondin-1,100 ng/ml Noggin	Primary mouse tissue
Schatoff Schatoff et al. (2019)	AdvDMEM/F12, penicillin/streptomycin, Glutamine, 1 mM N-Acetylcysteine, 10 U/ml DNAseⅠ, 1 ml FBS(final 5%)	Primary mouse tissue
Goto Goto et al. (2019)	AdvDMEM/F12, 50 ng/ml mouse EGF, penicillin/streptomycin, 10 mM HEPES, GlutaMAX, B27(1×)	Mouse
		CRC
Ganzleben Ganzleben et al. (2020)	AdvDMEM/F12, 10 nM HEPES, 2 mM GlutaMAX, Pen/Strep Amphotericin Mix, B27(1×), 1 mM Acetylcysteine, 50 ng/ml EGF	Mouse
		CRC
Sato Sato et al. (2011)	AdvDMEM/F12, penicillin/streptomycin, 10 mM HEPES, Glutamax, N2(1×), B27(1×), 1 mM N-acetylcysteine, murine EGF, murine noggin, human R-spondin-1, human Want-3A, gastrin, nicotinamide, A83-01, SB202190, Y-27632	Human
		CRC
Van de Wetering van de Wetering et al. (2015)	AdvDMEM/F12, 1% penicillin/streptomycin, 1% Hepes buffer, 1% Glutamax, 20% R-spondin-1 conditioned medium, 10% Noggin conditioned medium, B27(1×), 1.25 mM n-Acetyl Cysteine, 10 mM Nicotinamide, 50 ng/ml EGF, 500 nM A83-01, 10 μM SB202190, 100 μg/ml Primocin, 10 μM Y-27632	Human
		CRC
Fujii Fujii et al. (2016)	AdvDMEM/F12, penicillin/streptomycin, 10 mM HEPES, 2 mM GlutaMAX, B27(1×), 10 nM gastrin I, 1 mM N-acetylcysteine, 50 ng/ml mouse recombinant EGF, 100 ng/ml mouse recombinant Noggin, 10% R-Spondin-1 conditioned medium, 50% Wnt-3A conditioned medium, 500 nM A83-01, 10 μM SB202190	Human
		CRC
Buzzelli Buzzelli et al. (2018)	DMEM/F12, GlutaMAX, 1% StemPro, 10 ng/ml Y-27632, 100 ng/ml R-Spondin-1, 10 ng/ml Noggin, 10 ng/ml WNT3A, 10 ng/ml EGF, 5 ng/ml IGF-1, 10 ng/ml FGF-10, 10 ng/ml FGFβ, 10 ng/ml ET3	Human CRC liver metastases pathological specimens
Toden Toden et al. (2018)	AdvDMEM/F12, 50%(v/v) L-WRN conditioned medium, 20% FBS, 2 mM L-glutamine, 0.2% Primocen, 10 μM Y-27632, 10 μM SB431542, 5% penicillin/streptomycin	APC^Min^ mouse CRC and human CRC
Ubink Ubink et al. (2019)	AdvDMEM/F12, penicillin(100 U/ml), streptomycin(100 μg/ml), 10 mM Hepes, 400 μM Glutamax, B27(1×), 1 mM N-Acetyl-L-cysteine, 50 ng/ml Noggin, 500 nM A83-01, 10 μM SB202190	Human CRC peritoneal metastases pathological specimens
Ng Ng et al. (2019)	AdvDMEM/F12, penicillin/streptomycin, 10 mM HEPES, 2 mM Glutamax, B27(1×), 1 mM N-acetylcysteine, 10 nM gastrinⅠ, 50 ng/ml recombinant human noggin, 50 μg/ml recombinant human R-spondin-1, 500 nM A83-01, 10 mM SB202190, 10 μM Y-27632	Human
		CRC
Mukohyama Mukohyama et al. (2019)	AdvDMEM/F12, 2 mM GlutaMAX, 10 mM HEPES, 1 mM sodium pyruvate, 10% heat-inactivated FBS, 120 μg/ml penicillin, 100 μg/ml streptomycin, 0.25 μg/ml amphotericin-B, ITES mdia supplement, 50 ng/ml hEGF, 500 ng/ml hR-Spondin-1, 100 ng/ml hNoggin, 10 μM Y-27632	Cell lines and human CRC PDX
Lee Lee et al. (2019)	AdvDMEM/F12, B27(1×), 1.25 mM N-acetyl cysteine, 50 ng/ml human EGF, 50 ng/ml human Noggin, 10 nM gastrin, 500 nM A83-01, 100 mg/ml primocin, 10 μM Y-27632	Human
		CRC
Knight Knight et al. (2021)	AdvDMEM/F12, HEPES(5 mM), 2 mM L-glutamine, 100 U/ml penicillin/streptomycin, N2(1×), B27(1×), 100 ng/ml noggin and 50 ng/ml EGF, 500 ng/ml R-spondin, 10 nM gastrin, 100ng/ml Wnt-3A, 10 μM Y-27632, 0.5 μM A83-01, 5 μM SB202190, 4 mM nicotinamide, 10 ng/ml FGF basic, 10 ng/ml FGF10, 1 μM prostaglandin E2	Human
		CRC
Costales-Carrera Costales-Carrera et al. (2020)	AdvDMEM/F12, Hepes 10 mM, Glutamax 10 mM, N2(1×), B27(1×), 1 mM N-acetyl-L-cysteine, Primocin(1:500), 0.1 μg/ml Noggin, 1 μg/ml Gastrin, 10 μM Y-27632, 50 ng/ml EGF	Human
		CRC
Narasimhan Narasimhan et al. (2020)	AdvDMEM/F12, 10 mM Hepes, Glutamax(1×), 10 mg/L gentamicin, antibiotic-antimycotic(1×), B27(2×), 500 nM A83-01, 50 ng/ml hEGF, 1 nM Gastrin 1 human, 1 mM M-Acetyl-L-cysteine, 5 μM SB202190, 10 μM SB431542, 10 μM Y27632	Human
		CRC
Song Song et al. (2020)	AdvDMEM/F12, 50%(v/v)L-WRN-conditioned medium, Wnt3a, R-spondin, Noggin, penicillin/streptomycin(1×), 10 mM HEPES, 2 mM GlutaMAX, B27(1×), N2(1×), 1 mM N-Acetylcysteine, 10 nM Gastrin, 10 mM nicotinamide, 10 μM SB202190, 50 ng/ml recombinant murine EGF, 0.5 μM A83-01	Human
		CRC
Costales-Carrera Costales-Carrera et al. (2020)	AdvDMEM/F12, 10 mM HEPES, 10 mM Glutamax, N2(1×), B27(1×), 1 mM N-acetyl-L-cysteine, Primocin(1:500), 0.1 μg/ml Noggin, 1 μg/ml Gastrin, 10 μg/ml, 10 μM Y-27632, 50 ng/ml EGF, 0.02 μM PGE2, 1 μM LY-2157299, 10 μM SB-202190	Human
		CRC

## Application of Organoids in CRC Research

**FIGURE 1:**
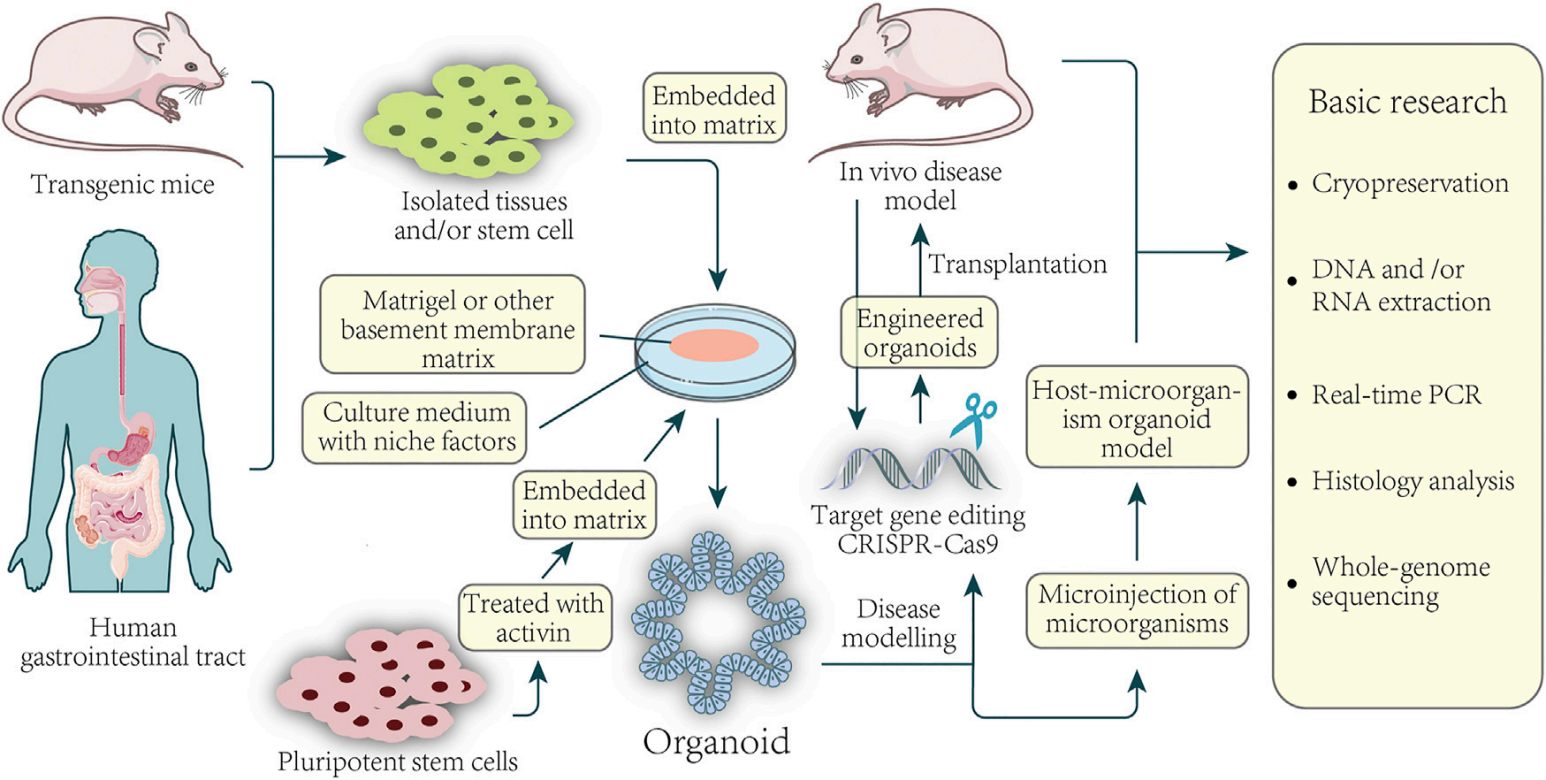
Basic application of colorectal organoids in disease and basic research. Organoids can be established from healthy or CRC tissue from the patient-sourced samples. Isolated tissue or stem cells, or activin-treated human pluripotent stem cells, are embedded in a basement membrane matrix and maintained in media containing a variety of niche factors critical for expansion. Organoids are usually formed in 3D and have structures similar to their origin. For example, culturing isolated gastric epithelial cells under the conditions described above can grow human gastric organoids in which gastric gland zone buds are distributed around the central lumen. Gene editing of organoids can be used for the modeling of diseases as well as the construction of xenografts. Direct sampling through mice and organoids can be used for a series of fundamental studies.

The intestinal organoid is the first organoid successfully cultivated, and it is also the most mature organoid produced and studied. Organoids are considered the best preclinical models, which can be used to construct disease models, tumor mechanism research, drug screening, tumor treatment and personalized medicine, drug development, regenerative medicine, etc. Organoids can culture, subculture, frozen storage, recovery, the genetic background, and histological characteristics are similar to internal organs. Organoids have been considered good preclinical models applied to many fields, such as construct disease models, tumor mechanism research, organoids construction of living organoids, drug screening, personalized drugs, novel drug development, drug toxicity, safety testing, and regenerative medicine. The potential applications of organoids of colorectal cancer patients are shown in Figure 1.

### Disease Model Construction

Organoids were first cultured to test the drug sensitivity of Orkambi in patients with cystic fibrosis (CF) and succeed (Sato et al., 2011; Dekkers et al., 2013), which might be applied to CRC. Sato (Sato et al., 2011) pioneered the development of intestinal organoids, and he isolated intestinal adenomas from APC-deficient mice and successfully formed cystic organoids *in vitro*. Moreover, he found that R–spondin–1 and Noggin, which are essential for normal small intestine organoid culture, were not necessary in adenomatous organoids. Then he applied the intestinal adenoma culture condition to human CRC samples and got CRC organoids with irregular dense structures, requiring neither R-spondin nor Noggin. Patient-derived organoids recapitulated the original tumor tissues’ histological features and marker expression, which also presented patient-specific heterogeneous morphologies and remained mutational spectrum observed in paired tumors in the most frequently mutated CRC genes with a 94.4% overlap (Yao et al., 2020). Weeber et al. (Weeber et al., 2015) obtained metastatic cancer tissues from 14 patients with CRC by a biopsy to culture organoids, of which 10 were successful. They then sequenced a total of 1977 cancer-related genes from tumor biopsies of eight patients and its matched biopsy-derived culture. They found that 90 percent of somatic mutations were shared between organoids and biopsies from the same patient. The correlation between the DNA copy number and the corresponding primary tumor was 0.89. Most importantly, none of the mutations found exclusively in the tumor or organoid culture are in driver genes or genes amenable to drug targeting. van de Wetering et al. (van de Wetering et al., 2015) generated 22 CRC organoids and 19 normal adjacent organoids from 20 patients. They proved that CRC organoids retained the subtypes of the original tumor samples by sequencing all exons. Buzzelli et al. (Buzzelli et al., 2018) proved that CRC liver metastases organoids show characteristics of colonic origin and different expression of stem cell markers, which could be screened for susceptibility to novel drug therapies. Janakiraman et al. (Janakiraman et al., 2020) observed that rectal cancer *in vitro* tumor organoid models replicated the clinical 5-fluorouracil chemotherapy with radiation neoadjuvant therapy pathologic tumor regression grading observed in the corresponding patient tumors. Therefore, the CRC organoid culture has a high success rate and reflects the genetic characteristics of patients, which can provide strong evidence for individualized treatment of cancer. In addition to producing organoids from patients with CRC, with the development of gene-editing technology, researchers have successfully introduced mutations into organoids derived from normal intestinal epithelium to produce CRC organoids for the study of diseases. Matano et al. (Matano et al., 2015) used CRISPR - Cas9 gene-editing technology to mutate a series of tumor suppressor genes APC, Smad4, TP53, and oncogenes KRAS, PI3K in normal epithelial organs, and produced corresponding CRC organoids via selective culture in medium without corresponding growth factors. At the same time, these modified organoids were transplanted into the kidney capsule and spleen of nude mice, which showed that they had different abilities of tumor formation, invasion, and metastasis, and indicated that they could reproduce the occurrence and development of CRC *in vitro*. Fujii et al. (Fujii et al., 2015) used the CRISPR–Cas9 system for precise genome editing. They obtained genetically modified human colonic organoids within 3 weeks through piggyBac transposon stable integration genes and efficient electroporation. Not content with culturing CRC organoids *in vitro*, researchers tried diseased organoids into mice and successfully established a model of CRC transplantation. Drost et al. (Drost et al., 2015) used CRISPR - Cas9 technology to modify the four most common CRC genes (APC, TP53, KRAS, and Smad4) in human intestinal stem cells and select mutated organoids by removing a single growth factor. Next, they subcutaneously injected organoid lines into mice. Some mice were injected with triple–mutant organoids (3 out of 12 injections). The majority of mice were injected with quadruple–mutant organoids (13 out of 16 injections) developed visible nodules. The quadruple mutants grew as tumors with features of invasive carcinoma. Roper et al. (Roper et al., 2017) injected CRC organoids with APC, TP53, and Kras mutations via CRISPR - Cas9 technology into the intestinal mucosa of mice to establish an orthotopic transplantation model. After 6 weeks, 15 tumors were found in 10 mice, and liver metastasis occurred in 3 mice after 12 weeks. Then, they injected human CRC organoids into the intestinal mucosa of mice. 8 of the 26 mice developed liver metastasis, and the resulting tumors highly maintained the morphological and pathological characteristics of the primary tumor. O'Rourke et al. (O'Rourke et al., 2017) induced colon injury to form a favorable microenvironment for tumor colonization. They injected the gene-edited mice organoids into the intestine of mice by enema to establish an immune CRC model. Fumagalli et al. (Fumagalli et al., 2018) surgically implanted CRC organoids into the cecum of mice, which resulted in tumor metastasis to the liver and lung with 100% efficiency. This model summarized the whole adenoma-adenocarcinoma-metastasis axis *in vivo*. This model is also applicable to orthotopic transplantation of patient-derived CRC organoids to produce a histopathological accurate preclinical human cancer model. Gastrointestinal tissue has been in a rhythmic peristaltic environment in the human body, and gastrointestinal organoids cannot simulate this environment. Recently, Guocheng Fang et al. (Fang et al., 2021) developed a microfluidic chip to imitate the rhythmic peristaltic culture of human colon tumor organoids. This chip consists of 200 lateral microwells and a surrounded pressure channel. Each microwell will grow into 1–2 colon tumor organoids that receive periodical contraction by the pressure channel and simulate the peristalsis. It was found that human colon tumor organoids grown in a peristaltic environment had a more uniform size distribution, and the expression of KI67 and LGR5 enhanced. Moreover, this chip achieves the high-throughput culture of organoids. Unexpectedly, the uptake of the ellipticine-loaded polymeric micelles by human colon tumor organoids grown in peristaltic environments was significantly reduced, which causes a reduction in antitumor activity. This study provides a new strategy for the large-scale dynamic culture of digestive organoids. The above-mentioned *in vitro* CRC organoids and organoid transplantation models have great potential in researching CRC drug screening, disease progression, and individualized treatment.

### Establishment of Living Biobanks of Organoids

**FIGURE 2:**
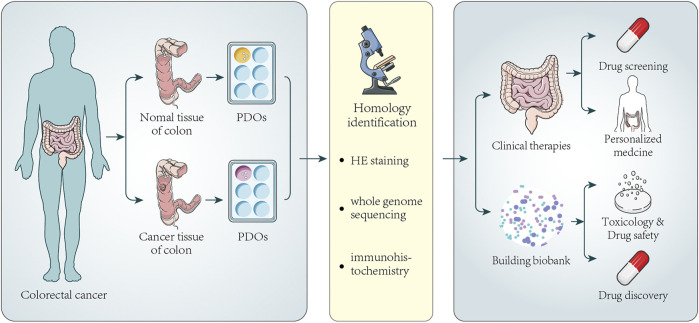
Establishment of colorectal cancer organoids and its application in clinical therapy and biobank. Tumor tissues and normal tissues of a certain number of colorectal cancer patients are collected to generate PDOs. Next, HE staining, immunohistochemistry, and whole-genome sequencing analysis are used to confirm that PDOs have similar histopathological features and genomic features to the original tumors. Part of PDOs can be directly used for drug screening and personalized medicine. The screening of new drugs always needs a large number of PDOs. Once a new drug appears, it can be used to test the potential activity and toxicity of new drugs, improving the speed and success rate of new drug development and promoting drug development. The other parts of PDOs can be stored to establish biobanks. The biobank contains a certain number of patient organoids, and the analysis of PDOs also makes it possible to compare drug sensitivity between individual patient responses.

Since various tumor organoids were successfully established, researchers from home and abroad have devoted themselves to establishing living biobanks of tumor organoids again. In recent years, many large-scale living organoid biobanks have been established abroad, including breast cancer (Sachs et al., 2018), gastric cancer (Yan et al., 2018), advanced prostate cancers (Beshiri et al., 2018), bladder cancer (Lee et al., 2018), metastatic colorectal and gastroesophageal cancer (Vlachogiannis et al., 2018), paragangliomas (Verginelli et al., 2018), glioblastoma (Jacob et al., 2020), tumors arising in the pancreas and distal bile duct (Driehuis et al., 2019), urothelial cancer (Mullenders et al., 2019), CRC (Kuipers et al., 2015; van de Wetering et al., 2015), and locally advanced rectal cancer (Yao et al., 2020). Figure 2 illustrates the information on the establishment and application of colorectal cancer organoid biobank. Establish a living biobank using tissue from patients. The aim was to screen suitable drugs for these patients. For the patient, the most obvious benefit is that drug testing can be performed without the patient’s participation. If there are no suitable drugs, the organoids would be kept in this organoid biobank. Once there is a new drug, they might test again to screen appropriate drugs. At present, living organoid biobanks are mainly used for drug screening, covering the genetic diversity of patients’ tumors, identifying specific drug-gene interactions, and providing more possibilities for personalized treatment of cancer patients. This work is inspiring, showing the powerful prospects of living organoids biobank in cancer biology research. Van de Wetering et al. (van de Wetering et al., 2015) successfully established a living biobank of CRC, including 22 tumor organoid cultures and 19 normal-adjacent organoid cultures derived from 20 patients. H&E staining of primary tumors and the corresponding organoids showed that “cystic and solid”-organization of the epithelium was generally preserved. Transcriptome analysis of individual organoids showed subtle differences in gene expression in organoids, confirming their heterogeneous composition. Genomic characterization demonstrates that organoids faithfully capture the genomic features of the primary tumor from which they derive and much of the genomic diversity of CRC, which also demonstrates that the gene change spectrum in the “living Biobank” is very consistent with the previous large-scale mutation analysis of CRC. RNA analysis indicates organoids in this living biobank of CRC represent the main molecular subtype of CRC. It is found that organoids closely summarized/recapitulated several properties of the original tumor, which are amenable to high-throughput drug screens and detect gene-drug associations. Fujii et al. (Fujii et al., 2016) established a tumor tissue living organoid biobank derived from 55 CRCs patients, including various tumor subtypes. They found that patients with different subtypes of CRC require different culture medium. In addition, different tumor mutation backgrounds need different culture medium components. It was also found that tumor tissue organoids retain the pathological characteristics of the original tumor tissue and can be xenotransplanted under the kidney capsule of immunodeficient mice. Georgios Vlachogiannis et al. (Vlachogiannis et al., 2018) used 110 fresh biopsy samples from 71 metastatic, heavily pretreated colorectal and gastroesophageal cancer patients recruited in four prospective phases I/II clinical trials to establish a living organoid biobank of CRC. Histological evaluation showed significant morphological similarities between PDOs and the patient biopsies. The immunohistochemistry markers routinely used in the diagnosis of CRC indicate that the expression pattern of patient tumors was still maintained in PDOs. Similarly, amplification of some oncogenic drivers was retained in PDOs. Genotypic profiling also revealed a high similarity between the original patient tumor and PDOs. CRC is a heterogeneous disease, and the clinical manifestations and prognoses of patients are different. Now, the treatment strategy of tumors is very diverse. It also did not turn out to be the curative effect we expected. The reason why tumors are difficult to treat is that it is closely related to the heterogeneity of the tumor. Therefore, it is possible to recapitulate inter- and intra-tumor heterogeneity through a large number of samples of the organoid biobank and a related personalized treatment strategy, which are mainly used to evaluate the application value of organoids in predicting personalized drug response and related therapies currently, as well as patient responses in clinical trials. Organoid biobanks can also be used for gene editing and other research, the development of new cell therapy strategies, and the development of new drugs; organoid biobanks also can be used to quickly verify the efficacy, safety, and new indications of new drugs and drugs that have entered the market, conduct research on rare diseases, and carry out clinical research projects.

### Cancer Mechanism Research

CRC organoids can simulate the pathological mechanism of intestinal malignant tumors and the role of mutations in different signaling pathways. In the 1990s, Fearon et al. (Fearon and Vogelstein, 1990) presented a model for the genetic basis of colorectal neoplasia, and most CRCs follow this pathway. In this model, malignant tumors occur because genes change according to the sequence of specific preferences and the total accumulation of changes, rather than their order with respect to one another, is responsible for determining the tumor’s biologic properties. Constitutive activation of Wnt signaling is usually found in early adenomas, so mutations in this pathway are thought to cause tumors. Progression to the cancer stage is related to activating mutations in the EGFR signaling pathway and subsequent inactivating mutations in the TGFβ and p53 pathways (Fearon, 2011). Drost et al. (Drost et al., 2015) knocked out the APC and P53 genes of organoids through CRISPR-Cas9 and then found that the deletion of APC and P53 was sufficient to obtain chromosomal instability and extensive aneuploidy. Fumagalli et al. (Fumagalli et al., 2017) demonstrated that early deletion of p53 is beneficial to tumor progression through triple mutation in human organoids. The initial APC and KRAS mutations drive effective proliferation and growth, while inactivation mutations in Smad4 prevent differentiation during tumor progression. After obtaining APC, KRAS, Smad4, and TP53 gene mutations, the global translation of intestinal organoids can be strongly enhanced (Smit et al., 2020). *Apc* mutation can cause uncontrolled Wnt activation, leading to tumorigenesis in most CRC patients (Szvicsek et al., 2019). In intestinal organoids, PPARD upregulates BMP7/TAK1 signaling to enhance the activation of β-catenin in intestinal epithelial cells (Liu et al., 2019), while β-catenin/TCF4 complex activates the expression of NRF3 mRNA (Aono et al., 2019), both of which promote tumor progression. Reischmann et al. (Reischmann et al., 2020) systematically compared the effects of BRAFV600E and P53R172H alone or in combination on oncogene naive organoids from the large intestine of knock-in mice and found that BRAFV600E and p53R172H cooperate in colorectal carcinogenesis by conferring survival signals, MEK inhibitor resistance, and invasive properties. BRAFV600E mutant organoid cultures survived treatment with TGFβ and induced EMT process (Fessler et al., 2016). P53 loss alone (Takeda et al., 2019) or QKI-5 (a transcriptional target of P53) decline in expression (Mukohyama et al., 2019) confers the metastatic ability to intestinal tumors. Nguyen et al. (Nguyen et al., 2020) found that ERBB3 (a member of the EGFR) kinase activities contribute to the outgrowth of epithelial cells under NRG1-β1 exposure, which is usually overexpressed in colon cancers. Hu et al. (Hu et al., 2020) found that Dachshund homologue 1 reduces the phosphorylation of Smad4 and inhibits the BMP pathway through its interaction with Smad, thereby promoting the formation and stemness of CRC organoids. He et al. (Li et al., 2020) generated paired organoids derived from primary tumors and matched liver metastases in the same CRC patients and showed that inducible knockdown of SOX2 attenuated invasion, proliferation, and liver metastasis outgrowth. In addition to studying gene mutations in CRC, organoids are also used to study the role of the tumor microenvironment in tumor development and metastasis. Tumor growth and degree of tumors malignancy are differentially regulated by different fibroblast subtypes under the influence of Wnt signals (Mosa et al., 2020). Rare pericryptal Ptgs2-expressing fibroblasts secreting PGE 2 provide a micro-niche conducive to activating the pro-tumorigenic Yap program in neighboring stem cells driving tumorigenesis in the presence of mutations (Roulis et al., 2020). FGFR-inhibition prevents the formation of organoids in primary CRC cells (Otte et al., 2019). Fibroblast-derived EVs carry active amphiregulin as EGF activity in the intestinal stem cell niche (Oszvald et al., 2020a), inducing the proliferation of CRC cells in EGF-dependent patient-derived organoids (Oszvald et al., 2020b). Tran et al. (Tran et al., 2020) proved that glutamine could over-activate Wnt signaling and prevented cell differentiation, while α-ketoglutamine could rescue the dryness induced by bottom glutamine and inhibit Wnt signaling to restore cell differentiation. However, signet ring cell carcinoma of the large intestine increased the uptake and breakdown of glutamine by upregulating SLC1A5 and GPT2 (Wang J et al., 2020). Zhao et al. (Zhao et al., 2020) used organoid models to study the metabolic phenotypes of cancer stem cells (CSCs) and differentiated cancer cells (non-CSC) in CRC. Then they found that non-CSC-originated lactate promotes self-renewal of CSCs and thus contributes to CRC progression. Besides, CRC organoids are also used to study the mechanism of tumor treatment. Liang et al. (Liang et al., 2019) found that Triptolide inhibited the transcription of Pol III by destroying the formation of TFIIIB, thus inducing the growth arrest of CRC cells. Buczacki et al. (Buczacki et al., 2018) found that itraconazole inhibited the Wnt signal through the nonstandard hedgehog signal, then caused G1 cell cycle arrest, and finally transformed the cell into global senescence. Deleting the Fbxw7 in CRC organoids induces a decrease in sensitivity to 5—FU (Lorenzi et al., 2016). Butyrate increases the sensitivity of CRC organoids to radiation via increasing FOXO3A transcriptional activity and inducing cell cycle arrest (Park et al., 2020).

### Drug Screening and Personalized Medicine

#### Accuracy of Organoids in Predicting Drug Response

As an *in vitro* individualized preclinical model, organoids have great potential in drug susceptibility testing of personalized medicines. At present, drug sensitivity tests have proved the value of organoids in personalized medicine. However, their potential in predicting patient clinical outcomes is unclear. Further prospective studies and clinical validation are needed to determine the feasibility of organoids.

Chemotherapy and radiotherapy are the standard treatments for locally advanced or metastatic CRC patients. Cheri A. Pasch et al. (Pasch et al., 2019) cultured organoids from 90 cancer patient-derived specimens (colorectal, pancreatic, lung adenocarcinomas, and so on) to test the sensitivity to chemotherapy (5-fluorouracil (5FU) and oxaliplatin with different concentrations) and radiation; the result indicated that using this technology could prospectively predict the treatment response of patients with metastatic CRC. PDOs test predicted the response to biopsy lesions in more than 80% of patients treated with irinotecan in metastatic CRC patients, without misclassifying patients who would have benefited from this treatment. It was indicated that PDOs could be used to identify nonresponders to standard-of-care chemotherapy in CRC, thereby preventing CRC patients from undergoing ineffective irinotecan-based chemotherapy (Ooft et al., 2019). A living organoids biobank was generated from biopsy samples of a chemo-refractory metastatic CRC patient. Then they evaluated the response to FDA-approved therapies, including cetuximab, regorarenib, and TAS-102. PDOs and matched biopsy samples carrying KRASG12D and BRAFV600E mutations did not respond to cetuximab. However, it is interesting that PDOs with genotypic characteristics did not respond to cetuximab, which consisted of the patient’s results, indicating the predictive utility and accuracy (Bradley, 2018). 21 different rectal cancer (RC) organoids were separately treated with 5-FU and FOLFOX (a chemotherapy regimen of 5-FU, leucovorin, and oxaliplatin), and 19 RC organoids were received radiation treatment. The results showed that the RC organoids are sensitive to their response to doses of 5-FU and FOLFOX. They also achieved different sensitivity responses to radiation, corresponding to clinical chemoradiation responses (Ganesh et al., 2019). Recently, a blinded study using patients of stage IV colorectal cancer-derived tumor organoid was employed to evaluate the predictive accuracy for responses to chemotherapy regimens (Wang et al., 2021). In this blinded study, 71 patients met the inclusion criteria, 77 organoids were successfully established, achieving an 80.21% success rate. It was found that organoids and metastatic tissue were remarkably consistent with the original cancer tissue through identification. H&E staining showed that the organoid retained the histological characteristics and marker expression of the original tumor tissue. The results showed that the sensitivity, specificity, and accuracy of the tumor organoid model to predict the response of chemotherapy regimens were 63.33, 94.12, and 79.69%, respectively. Briefly, the patient-derived organoids can be used as a predictive model to effectively predict the drug response of personalized cancer patients and guide the selection of clinical personalized therapeutic regimens.

#### Drug Susceptibility Testing and Individualized Treatment

Drug sensitivity and individualized treatment are the most mature field of organoid applications. Organoids derived from patients retain corresponding features of tumors of patients. Its response to drugs is highly similar to the clinics (Puca et al., 2018). For patients, the most suitable drug can be quickly detected through organoids-drug sensitivity testing technology, and the best and most effective drug treatment plan can be formulated, reducing drug side effects, the drug resistance, and the probability of tumor recurrence, thereby obtaining the best therapeutic way. Organoids especially bring hope to patients with advanced drug resistance. Organoid models can be used to analyze the causes of drug resistance to find the next effective treatment strategy. Moreover, organoids are used for commonly used drugs sensitivity testing such as chemotherapeutics, targeted drugs, new antitumor antibody drugs, and for testing radiotherapy sensitivity *in vitro* and other therapies.

Cystic fibrosis transmembrane conductance regulator (CFTR) gene mutation causes CF (Moran, 2017). Using the cystic fibrosis patient-derived organoids model for drug screening and individualized treatment (Berkers et al., 2019) is the first clinical application case of using organoids to guide individualized medication. Johanna F Dekkers et al. used rectal organoids derived from CF patients to test the response of different CFTR-modulating drugs (Dekkers et al., 2016). Kalydeco was considered highly effective and wonder drug for CF patients. However, it was expensive and did not work for all CF patients. Hence, the researchers established intestinal organoids derived from CF patients to test a person’s response to kalydeco quickly and efficiently (Saini, 2016). It was finally determined that the patient population would benefit from specific types of CFTR-modulating drugs, then developed individualized treatment plans for patients. At present, organoid culture has become a routine diagnosis and treatment procedure for CF patients in the Netherlands. Up to August, 12 CF patients have been successfully cured by this method, and this is also the first case of applying the rectal organoid model to guide clinical treatment.

Georgios Vlachogiannis et al. (Vlachogiannis et al., 2018) established the patients-derived organoids living biobank and simulated the treatment response of targeted agents or chemotherapy of colorectal and gastroesophageal cancers. The results showed that organoids had a sensitivity of 100%, a specificity of 93%, a positive predictive value of 88%, and a negative predictive value of 100% in predicting the effectiveness of anticancer drugs. It was the first study that used patient-derived organoids to predict anticancer drug sensitivity, which indicated that CRC organoids could apply to drug sensitivity testing and personalized medicine. Pauli et al. (Pauli et al., 2017) combined the gene mutation data obtained by whole-genome sequencing of tumors in colon cancer patients with advanced disease and CRC organoids living biobank to screen corresponding targeted drugs and explore the effectiveness of drug combination applications. The results showed that the combination of afatinib and vorinostat significantly inhibited tumor growth in mice transplanted with tumor organoids with APC mutations, and the tumor volume was only 10% of that treatment with FOLFOX chemotherapy. Van de Wetering et al. (van de Wetering et al., 2015) successfully generated a living biobank of 22 colon cancer organoids. Combined with drug sensitivity experiments, they observed that IWP-2, a small molecule inhibitor of the O-acyltransferase Porcupine, has a strong inhibitory effect on the growth of organoids with RNF43 mutation. In contrast, the growth of organoids without RNF43 mutation is not affected. It could be considered a new target to treat colon cancer. Jarle Bruun et al. (Bruun et al., 2020) made drug sensitivity testing of 40 clinically relevant drugs and gene expression profiling with PDOs from 38 CRC liver metastases of 22 patients. There were significant differences in the sensitivity of patients to several anticancer drugs that were not approved for treatment of CRC, antimetabolites (gemcitabine and methotrexate), and some targeted drugs without clear genomic markers (alisetidine b and navitoclax) showed greater heterogeneity in anticancer activity than most approved chemotherapy drugs.

### Drug Discovery

Drugs sensitivity and treatment strategies were tested with the variety of patient-derived tumor organoids with genetic diversity and tumor heterogeneity, and the results were highly consistent with the clinical response of the patient to the corresponding drugs and treatment strategies (Pauli et al., 2017; Hill et al., 2018; Lee et al., 2018; Tiriac et al., 2018; Vlachogiannis et al., 2018). Patients-derived tumor organoids are more sensitive to cytotoxic drugs and can better predict patient drug safety (Pauli et al., 2017; Tiriac et al., 2018; Vlachogiannis et al., 2018). Patients-derived tumor organoids or living biobanks can also be used to screen efficient antitumor drugs of individuals and even for large-scale drug screening and discovery. It is an efficient model to screen and predict effective drugs for patients with tumor in a more accurate, quick, and safe way.

#### New Drug Discovery

For new drug discovery, organoids and their living biobanks can be used as the best models *in vitro*. Organoids and their living biobanks can significantly shorten the cycle of preclinical trials, reduce development costs and risks, increase the success rates, reverse the current situation that lacks excellent preclinical models in new drug discovery, and provide the best quality platform for new drug discovery. Breaking the original new drug discovery process will be a great benefit for new drug discovery companies and everyone. Plocabulin is a new marine original antitumor drug that destroys microtubules and undergoes phase II clinical trials. SN38 is an active irinotecan derivative. Alba Costales-Carrera et al. found that the cytotoxicity of plocabulin was much more efficient than SN38 in CRC organoids in different passages (Costales-Carrera et al., 2019). This experiment indicated that plocabulin has intense cytotoxic action in personalized CRC patient-derived tumor organoids, providing supplements for plocabulin clinical trials and accelerating the development of new drugs. In addition, organoids also have excellent potent in developing cancer immunotherapies. Patient-derived CRC organoids can be used to evaluate CAR-mediated cytotoxicity (Schnalzger et al., 2019). In recent years, many pharmaceutical companies have begun to invest many resources to build organoid platforms and organoid biobanks to promote the drug development process.

#### Drug Repositioning

Drug discovery is a time-consuming, high-investment, high-risk, and high-failure process in traditional drug development. 90% of new drugs cannot enter clinical trials because of preclinical data flaws, and more than a quarter of drugs fail due to lack of efficacy (Arrowsmith and Miller, 2013; Cook et al., 2014). The reason may be that there are some differences between the preclinical models used in the development of new drugs and humans. So, conventional drugs in new use are becoming an attractive option for cancer therapy. Currently, the success rate of drug repositioning has accounted for approximately 30% of FDA newly approved drugs and vaccines. The number of new drugs approved by the FDA is approximately 40 each year, and the success rate is less than 6% of new drug development (Pillaiyar et al., 2020). Daniel Eduardo Gomez et al. (Armando et al., 2020) summarized drug repositioning of many drugs in oncology, such as artesunate, auranofin, benzimidazole derivatives. Currently, there are usually three methods of drug repositioning: computational methods, biological experimental methods, and hybrid methods. Organoids and the corresponding human organs have highly similar histological features, can reflect the characteristics of the primary tumor, and can accurately predict the response of drugs. Hengli Tang et al. used organoids to screen the effect drug from 6,000 compounds with drug repurposing, which inhibited Zika virus infection and induced neural cell death (Xu et al., 2016). Suppose tumor organoids or organoid biobanks will be introduced into drug repositioning for high-throughput drug screening. In that case, the preclinical data will be more scientific and practical, significantly increasing the speed of new drug development and the success rate.

#### Drug Toxicity and Safety Evaluation

Adverse effects of drugs, especially organ toxicity, are the main reasons for drug development failure and withdrawal after marketing (Jamal et al., 2019). The current general cell screening and animal model screening often cannot accurately predict adverse reactions in the human body. Drug toxicity testing is another application of organoid technology to drug development. Many anticancer drugs mainly cause damage and toxicity to patients’ vital organs such as the heart, kidney, and liver (Han et al., 2017). Some drugs with unpredictable toxicity to the liver cause liver failure in some patients. Drug-induced liver failure is the main reason for the failure of drugs in clinical trials (van de Wetering et al., 2015). Human liver organoids were used to test the toxicity of aspartic acid-coated magnesium oxide nanoparticles and valproate. These two drugs have toxic effects on the liver organoids by reducing cell viability, reducing ATP, and increasing reactive oxygen species (Mekky et al., 2021). Liver organoids were applied to verify the toxicity of drugs that had been withdrawn from the market due to hepatotoxicity, such as troglitazone, trovafloxacin. The result indicates that liver organoids, as a potential model, could be used to test and evaluate drug toxicity (Mun et al., 2019). Kidney organoids were treated with gentamicin and cisplatin to detect the toxicity, and the result showed that gentamicin injured proximal tubules, cisplatin has proximal and distal tubular toxicity (Morizane et al., 2015). Takasato et al. (Takasato et al., 2015) also used human kidney organoids to verify the nephrotoxicity of cisplatin and gentamicin; the results explained the kidney toxicity of cisplatin and gentamicin. Cells isolated from mouse kidney tissue were combined with microfluidic techniques to create kidney organoids with tubular and glomerular structures. These kidney organoids were used to evaluate the biosafety of various quantum dots nanomaterials. Black phosphorus quantum dots have moderate toxicity (He et al., 2020). In addition, heart organoids also can be used to detect drug cardiotoxicity (Eder et al., 2016). Dylan J Richards et al. used heart organoids to model hypoxia-enhanced doxorubicin cardiotoxicity. The result showed that hypoxic cardiac injury aggravates the cardiotoxicity of doxorubicin (Richards et al., 2020). Using organoid biobanks, including large samples to detect the toxicity of drugs, can obtain more scientific and practical data. The organoid model plays a vital role in drug toxicity screening.

### Regenerative Medicine

Organ transplantation faces the problem of donor shortage and immune rejection. Researchers have been committed to solving this problem through regenerative medicine. If the patient’s stem cells are cultured *in vitro* to establish organoids and then transplanted back into the body, which will be an essential measure to solve this problem. The stem cell population reproduced from organoids can replace damaged or diseased tissues, playing a corresponding role. In the future, this technology also has great potential in regenerative medicine.

A single adult Lgr5+ stem cell can successfully expand into colon organoids *in vitro*. Transplanting colonic organoids into mice models of acute colitis induced by dextran sulfate repairs the damaged colonic epithelium by integrating it into the mouse colon to cover the area lacking epithelium due to injury, thereby repairing its damaged colonic epithelium (Roper et al., 2017). The transplanted colonic organoids constituted a single-layered epithelium, self-renewing crypts that normal function and histology 4 weeks after transplantation, showing the feasibility of colon stem-cell therapy based on the *in vitro* expansion of a single adult colonic stem cell. Sugimoto et al. (Fujii et al., 2020) established normal human colon organoids, transplanted them into the submucosa of the mouse colon *in situ*, and observed their growth in mice. It was found that the average human colon organoids are significantly different from that of mouse colonic crypts after transplantation, which demonstrates the self-renewal and multipotency of LGR5+ colonic stem cells in the mouse colon and reflects corresponding physiological functions that were generated after colonic organ transplantation. Toshiro Sato et al. (Lindemans et al., 2015) reported their advanced study of a new strategy to use organoids to treat intestinal failure. Integrating intestinal organoids derived from biopsy samples of children patients into intestinal stents to generate hybrid intestines (research found that organoids and decellularized intestinal stents are combined), which can absorb nutrients and has durability, elasticity, and epithelial lining of the graft. Surprisingly, it can survive for 2 weeks when transplanted under the kidney or subcutaneously in mice. It will open a new way to regenerate patients with intestinal failure. Thierry Jardé et al. (Jardé et al., 2020) reported that mesenchymal-derived neuregulin 1 (NRG1) could induce a proliferative gene signature, promote progenitor cells to form organoids, and enhance regeneration after injury. NRG1 is an effective mediator of tissue regeneration and contributes to intestinal repair therapy following injury by organoids technology.

Human intestinal organoids were applied to the research of some cytokines on the repair of following intestinal injury, recombinant interleukin-22 directly targets the intestinal stem cells, which enhances the growth of mouse and human intestinal organoids, increases proliferation, and promotes the expansion of the intestinal stem cells (Lindemans et al., 2015). In addition, some other organoids with physiological functions of the corresponding organ were successfully constructed *in vitro*. Molly E Kupfer et al. (Kupfer et al., 2020) combined organoid technology and 3D printing technology to produce a human heart organoid for the first time that can keep regular operation. Kazuki Takeishi et al. used human skin cells to induce induced pluripotent stem cells (iPSC) and successfully created liver organoids that secrete bile acid and urea like normal livers. They were transplanted into five rats and generally survived for 4 days (Takeishi et al., 2020). Transplanting organoids cultured *in vitro* back into mice and organoids can cover damaged or diseased tissues like band-aids and play an influential role in repairing. These studies are proof of the concept of potentially revolutionary technology, providing a glimmer of hope for saving the lives of patients with organ failure. This technology also has great potential in regenerative medicine and organ transplantation in the future.

## Limitations and Challenges

Owing to the development and expansion of the organoid culture system, organoid research has made significant progress. As a 3D culture model, organoids are being used in many types of researches, but its technology still has many limitations and challenges ahead. There are still many problems that need to be solved urgently. At present, organoids do not have the microenvironment of the human body and lack the necessary components in human tissues, such as the vascular system, nervous system, and immune system. The construction of a patient-derived tumor organoid model requires fresh tumor tissue from the patient. Nevertheless, some fresh tumor samples are difficult to obtain, such as ovarian cancer. The culture condition of tumor organoids is varied and complex. Different tissues of the same patient, even different subtypes, and the same tissue of different patients are different in culture conditions. Different laboratories generally use distinct culture conditions. The results obtained from different laboratories using organoids are also different. If different results are received, the selected drug may not have reasonable specificity, implying the importance of standardized preparation of tumor organoids. The problems mentioned above affect the construction efficiency and success rate of organoids. More reasonable culture methods need to be further studied. The standardization of organoids, which contribute to promoting the development of organoids and benefit human health, are the urgent problems that need to be solved.

There has been tremendous progress in studying organoids in the past 10 years, and organoids are being used in many kinds of research. However, there are still many deficiencies in the early development stage of organoid technology. On the one hand, scientists have usually only used iPSC to culture-specific cells and organs individually. Integrating multiple organs in stem cell culture is a crucial unsolved challenge. On the other hand, most intestinal organoids are derived from the intestinal crypt. The LGR5^+^ intestinal stem cells (ISCs) are self-renewal stem cells, which reside at the bottom of the crypt. These ISCs are divided into transport expanded cells and rapidly proliferating transit-amplifying (TA) cells. Intestinal epithelial cells, goblet cells, and intestinal endocrine cells are developed from TA cells (Barker et al., 2007). Although the crypt can simulate the function of intestinal organoids *in vitro*, it lacks vascular, neural, and immune cells and epithelial (stromal/mesenchymal) cells (Sato et al., 2009). Clevers et al. also indicated that current organoids have obvious limitations, such as the absence of innervation, blood vessels, and immune cells. Therefore, the disease process can only be partially reproduced (Clevers, 2016). Similarly, intestinal organoids cannot completely simulate the occurrence and development of intestinal tumors in the body, such as invasion and metastasis.

In addition, changes in gene expression are inevitable during the culture of CRC organoids. CRC is a heterogeneous disease involving various genomic and epigenetic changes (Lau et al., 2020). Some articles have shown that the cloned established cancer cell lines are highly genetically heterogeneous—the heterogeneity results from the number of existing subclones and the emergence of new genetic variations. Genetic heterogeneity leads to different gene expression patterns, which leads to different drug sensitivity (Ben-David et al., 2018), and some tumor organoids may not maintain clonal proliferation. However, some studies have shown that some mutations in gut organoids do not affect the differentiation of stem cells (Cao et al., 2015). Organoids have been genetically and phenotypically stable for several years by passaging at a ratio of 1:5 per week (Sato and Clevers, 2013). Heterogeneity and stability are some of the technical bottlenecks of tumor organoids, and its solution also requires the establishment of standards.

Matrigel is necessary for organoid culture, which is three-dimensional lamin and rich in collagen. It is generally impossible to establish a long-term culture from primary adult tissues without inducing genetic transformation (Sato and Clevers, 2013). Most of the matrigel used in organoid culture is animal-derived. In the future, if we transplant organoids into the human body, it will also involve ethical issues.

At present, drug sensitivity analysis is the most mature application of organoids. Tumor-tissue specimens from biopsies, tumor resections, or ascites could be used to culture patient-derived organoids. Then drug sensitivity analysis is performed to screen out the most effective drugs to guide personalized clinical treatment. However, the organoids of some cancer patients not only grow slowly but also have a low success rate, especially in some advanced patients and cancers with a high degree of malignancy. Therefore, as an effective drug-screening model, it is also worth exploring how to improve existing organoid construction efficiency and success rate. The core problem is the lack of standards in the field of organoids. The multi-model combination also is an alternative solution to these limitations.

## Future Directions

The challenges that organoids face today also let us clarify the future development direction of organoids. Compared with traditional technology, we always believe that organoids have their unique advantages, which will significantly promote the development of basic research and clinical treatment of cancer. Some researchers have cultured liver organoids with blood vessels (Sato and Clevers, 2013), and others have migrated neural stem cells (NCC_S_) into human intestinal organoids mesenchyme. NCCs can differentiate into neurons and glial cells, which support neuronal activity (Workman et al., 2017). Therefore, we could build organoid models with the tumor microenvironment, vascular system, and nervous system only when we overcome these challenges as mentioned above. This organoid model will be closer to the human body or even build more efficient multiorgan integration models, combined with microfluidic technology, gene-editing technology, live-cell imaging, and other technologies. Organoid technology will greatly promote human health in the future.

### Construct the Tumor Microenvironment

The tumor microenvironment (TME) is the critical factor for tumor cells to occurrence, growth, proliferation, and metastasis; meanwhile, it affects the structure, function, and metabolism of tumor tissue (Hu et al., 2018; Du et al., 2020; Wang R et al., 2020). More and more evidence shows that the malignant behavior of cancer is caused by cancer cells and is fundamentally affected by the activity of the cancer stroma which includes various mesenchymal cells, especially fibroblasts, myofibroblasts, endocrinocytes, pericytes, inflammatory cells, and extracellular matrix. Moreover, researches also showed that the malignant of cancer cells is controlled by the microenvironment through various mechanisms (Yuan et al., 2016; Bu et al., 2019). Stromal cells can support tumor growth, metastasis, and drug resistance (Lau et al., 2020). For colon cancer cells, the extracellular matrix can promote the growth of colon cancer cells and blood vessels (Romero-López et al., 2017). The vast heterogeneity within cell types of the TME critically affects treatment responses (Junttila and de Sauvage, 2013). In terms of drug research and development, the interaction between cancer cells and stromal cells can enhance the responsiveness of cancer cells to the drugs and contribute to drug resistance (McMillin et al., 2010). Cocultivation of organoids with other types of cells is a new research hotspot in organoids in recent years, including immune cells, tumor cells, intestinal cells, and mesenchymal cells, which provides another way to simulate the better development of tumor organoids. Currently, two methods can be used to construct a tumor immune microenvironment in organoids, and native immune TME can be simulated by PDO gas-liquid interface (ALI) or PDO tumor microfluidic device. TME can be reconstructed by adding a purified immune population to submerged tumor epithelial organoids. Organoid established by the method of ALI builds TME retains tumor parenchyma and stroma of primary tissues and contains tumor-specific tumor-infiltrating lymphocyte populations. In addition, both mouse and human tumor tissues have verified that ALI-PDOs can restore PD-1-dependent immune checkpoints. Building a personalized organoid model with TME could contribute to a better tumor immunotherapy (Neal et al., 2018). Krijn K Dijkstra et al. demonstrate that cocultures of autologous tumor organoids and peripheral blood lymphocytes can enrich tumor-reactive T cells from peripheral blood of patients with mismatch repair-deficient CRC and non-small-cell lung cancer. Moreover, these T cells can also be used to evaluate the efficiency of killing matched tumor organoids (Dijkstra et al., 2018). Chiara M. Cattaneo et al. performed an initial coculture of 2 weeks of tumor organoids and autologous peripheral blood lymphocytes from patients with nonsmall cell lung cancer (NSCLC) and microsatellite unstable CRC. They found that tumor-reactive CD8^+^ T-cell populations can be tested from 33 to 50% of samples from patients. In addition, the evaluation of tumor-reactive T-cell function found that it was possible to establish an *ex vivo* test system for T-cell-based immunotherapy at the level of an individual patient (Cattaneo et al., 2020). Mouse colon cancer organoids are cocultured with adipocytes, and the result indicates that adipocytes in the TME serve as an energy provider and a metabolic regulator to promote the growth and survival of colon cancer cells (Wen et al., 2017). In addition, a microfluidic chip is also a way to generate the TME. Hypoxia is an essential feature of the tumor microenvironment. Lulu Zheng et al. have developed a microfluidic chip that involves lung cancer and liver organoids. By precisely regulating the oxygen concentration in each chamber and monitoring the oxygen concentration level with sensors on the chip, the anoxic environment is achieved in the lung tumor organoids chamber of the upstream. The downstream chamber imitates the oxygen environment of the normal liver organoids, which realizes the cancer metastasis induced by the oxygen-deficient environment on the chip and can be used to the antitumor drug screening under the oxygen-deficient environment (Zheng et al., 2021). More and more tumor organoids generate the TME by microfluidic chips and coculture technology. Organoid, an *in vitro* tumor model, will be applied increasingly to simulate *in vivo* tumors.

### Vascularization Research in Organoids

To a certain extent, organoids can reproduce the specific 3D structure and certain physiological functions of tissues or organs in the human body and show their unique advantages in many aspects, so many researchers are pinning their hopes on the new technology. However, the currently cultivated organoids cannot wholly simulate actual tissues and organs regardless of their shape, size, or physiological functions; it can only simulate tissues and organs in the human body under impact to a certain extent. At present, organoids cultured *in vitro* often lack effective blood vessels. In the process of organoid culture, as the volume of organoids increases, hypoxia and accumulation of metabolic waste products will lead to cell apoptosis. The vascularization of organoids has always been a hot and challenging point in organoid research, which is one of the major bottlenecks in organoid culture technology, which is also related to the development of organoids in the future.

At present, only a few types of vascularized organoids have been successfully produced, such as brain organoids (Mansour et al., 2018; Pham et al., 2018; Cakir et al., 2019; Shi Y. et al., 2020; Ham et al., 2020), liver organoids (Baptista et al., 2011; Rennert et al., 2015), pancreatic organoids (Choi et al., 2021), kidney organoids (Choi et al., 2021), and intestinal organoids (Holloway et al., 2020). Endothelial cells play essential roles in angiogenesis, vascular and tissue remodeling (Shu et al., 2014). However, adult endothelial cells do not vascularize tissues in an organotypic manner. Brisa Palikuqi et al. transiently reactivated embryonic restricted ETS variant transcription factor 2 (ETV2) immature human endothelial cells cultured in a serum-free 3D matrix. Then they reset these endothelial cells to adaptive angiogenic cells, which form perfusable and plastic vascular plexi. The “reset” vascular endothelial cells (R-VECs) vascularize the decellularization of rat intestines and arborize colon organoids of healthily or cancer patients (Palikuqi et al., 2020). Continuous delivery of VEGF will upregulate microvasculature and epithelial proliferation in the tissue-engineered intestine (Rocha et al., 2008). Emily M. Holloway et al. found that a population of endothelial cells (ECs) present early in human pluripotent stem cell (hPSC)-derived intestinal organoids (HIO) differentiation that declines over time in culture. Recently, they added EGF, VEGF, BFGF, and BMP-4, and other cytokines to the culture system based on conventional intestinal organoid culture methods to induce co-differentiation of endogenous ECs to form vascularized small intestinal organoids (vHIOs) (Holloway et al., 2020). After several weeks of culture *in vitro*, immunofluorescence assay results showed many CD31/CD144 double-positive ECs cells in the interstitium of vHIOs, which confirmed the enrichment of endogenous ECs. Moreover, ECs can survive for 2 months in the culture system. Shravanthi Rajasekar et al. designed a microfluidic platform named IFlowPlate, which successfully cocultured patient-derived colon organoids in a network of self-assembled vascular by optimizing the extracellular matrix (ECM) and formulation of culture media. This platform can be used to culture up to 128 isolated perfused and vascularized colon organoids *in vitro*. A colon inflammation model with the innate immune function was developed using this platform. The IFlowPlate platform also opens up new possibilities for screening potential therapeutic targets or modeling-related diseases based on the ability to grow vascularized colon organoids with intravascular perfusion (Rajasekar et al., 2020). Blood vessels are critical for the construction of atherosclerosis models. iPSC-derived cells are loaded into microfluidic devices and exposed to atherosclerotic stimuli to mimic atherosclerosis and build the atherosclerosis model *in vitro*. The study is the first to simulate the development of arteriosclerosis plaques *in vitro* fully (Mallone et al., 2021). The decisive factors of organoid angiogenesis are not apparent, but the conditions and culture with different organoids. Microfluidics may be a solution for the vascularization of organoids. The vascularization of organoids needs further research. As researchers continue to deepen, scientists will successfully construct organoids with blood vessels *in vitro* soon.

### Restructuring Intestinal Flora

There is a dynamic balance between intestinal microbes and human immune cells. Once the balance of intestinal flora is broken, it will cause a series of inflammatory reactions and immune system diseases and even lead to cancer. The composition and functional status of different members of the microbial community can modulate or even control cancer initiation, progression, comorbidity, and response to therapy (Dzutsev et al., 2017). It has been shown that some intestinal microorganisms can promote or inhibit the development of intestinal tumors (Tilg et al., 2018). Studies have shown that *Enterococcus faecalis*, *Streptococcus bovis*, and *Fusobacterium* in patients with CRC are significantly higher than in normal persons (Kramer and Genco, 2017). Preoperative supplementation of viable *Bifidobacteria* for patients with CRC can reconstruct the balance of intestinal flora and reduce infection complications of surgery (Zhang et al., 2010). Inosine is a metabolite of *Bifidobacterium pseudolongum*, *Lactobacillus johnsonii*, and *Olsenella*, which can effectively activate the antitumor T cells, shrink the volume of tumors, and even eliminate all CRC cells in some cases (Mager et al., 2020). In addition, intestinal flora also affects the efficiency of chemotherapy and radiotherapy (Iida et al., 2013; Touchefeu et al., 2014). In short, the intestinal flora plays a vital role in the occurrence, development, and treatment of CRC. InsP3, a microbiota-derived metabolite, induced growth of intestinal organoids derived from human tissue, facilitated the repair of intestinal damage, promoted the HDAC3 activity in the gut, stimulated HDAC3-dependent proliferation, and countered butyrate inhibition to colonic growth (Wu et al., 2020). Human intestinal organoids were repeatedly injected with genotoxic pks+*Escherichia coli* culture for 5 months (Pleguezuelos-Manzano et al., 2020). Whole-genome sequencing of clonal organoids was performed before and after this exposure of isogenic pks-mutant bacteria and revealed the distinct mutational signature absent from organoids injected with isogenic pks-mutant bacteria. Human ileal organoids grown under four different culture conditions were exposed to SARS-CoV and SARS-CoV-2 with the multiplicity of infection. Then they harvested these samples at multiple time points postinfection and performed related tests, and they found that enterocytes were more easily infected by SARS-CoV and SARS-CoV-2 (Lamers et al., 2020).

### The Integration of Multiple Organs

The human body is an organic whole, and there are information transmission and a number of interactions between cells, tissues, and organs (Xu et al., 2019). For a long time, scientists could only cultivate a particular type of organoids, and the integration of multiple organs is a crucial challenge in organoid research. However, organoids are used as an *in vitro* model to better human health and make research results more accurate and reliable. Moreover, the integration of multiple organoid models is a trend in the future. Remarkably, it is for the first time forms the boundary interactions between the anterior and posterior gut spheroids, differentiated from human pluripotent stem cells and enabled autonomous emergence of three kinds of interconnected organoids domains specified at the foregut-midgut boundary without the absence of supplement of extrinsic factor, including liver, pancreas, and bile duct (Koike et al., 2019). The microfluidic chip can also be used for the construction of multi-organoid models. The microfluidic chip of lung tumor organoids and liver organoids linkage model has been successfully constructed and conducted drug screening at a multi-organ level (Zheng et al., 2021). In the future, further studies are needed for the integration model of multiple organoids, which will better contribute to the development of new drugs and drug toxicology.

### Combination With Other Cutting-Edge Biomedical Technologies

Organoids can be combined with other cutting-edge biomedical technologies to display its better properties in different researches. Organoids can be combined with microfluidic technology to imitate the *vivo* tumor microenvironment (Schuster et al., 2020). Organoids and organ chip technology (Park et al., 2019; Cong et al., 2020) can be used together to mimic the microenvironment of organoids and establish mutual relationships between tissues and multiple organs, and it also can reduce the randomness and variability in the culture process. Furthermore, it was reported that organoids coupled with the microfluidic chip technology to get meaningful drug responses within a week, which is a good way for saving time, enabling patients to receive effective treatment earlier, and prolonging life (Hu et al., 2021). Organoids can be combined with live-cell imaging technology (Bolhaqueiro et al., 2019) to study the biological mechanism by monitoring the process. Organoids also can be combined with single-cell technology (Chen et al., 2018; Wang Y et al., 2020) to identify whether tumor organoids can summarize the heterogeneity of tumor patients and understand the occurrence and development of diseases through organoid models. In addition, organoids can be combined with CRISPR gene-editing technology (Roper et al., 2017; Artegiani et al., 2020; Ringel et al., 2020). This efficient organoid gene-editing system can be used to study the molecular mechanism of colon cancer development, quickly characterize cancer-related genes *in vivo*, reproduce the entire process of tumor progression and metastasis, and study its mechanism. Organoids can also be used with high-content imaging technology to study the mechanism of drug action (Brandenberg et al., 2020) and evaluate the liver toxicity of drugs (Joshi et al., 2018; Peel et al., 2019). David A Sinclair et al. used liver organoids that infected with SARS-CoV-2 and artificial intelligence (AI) to prove that Baricitinib can improve the condition of four COVID-19 patients (Schultz et al., 2020). AI analysis is introduced into organoids to assess drug effects and new drug development (Abdul et al., 2020). Organoid technology, microfluidics, and 3D printing technology are combined to quickly establish an automated organoid platform, which is expected to meet the goal of one-week high-throughput screening of drugs for cancer patients in 1 week and personalized medicine (Jiang W et al., 2020). Organoids have broad prospects; it can be used in conjunction with cutting-edge biomedical technologies and can play a better role in research.

### Organoid Standardization

With the rapid development of organoid technology, the standardization of organoids urgently needs to be solved. There are some problems in the organoid culture process, which will affect the application and the development of organoid technology. Therefore, we mainly discuss the standardization of organoids from the aspects of Matrigel, maturation of organoids *in vitro*, standardization of operating procedures, and organoid definitions.

Matrigel, a tumor-derived extracellular matrix with 3D lamin and rich in collagen, is necessary for organoid culture. Most of the Matrigel used to organoids culture is animal-derived. In the future, if we transplant organoids into the human body, it will also involve ethical issues. Victor Hernandez-Gordillo et al. completely synthetic a new tumor-derived extracellular matrix for *in vitro* culture of primary human intestinal enteroids and endometrial organoids. They found that this new Matrigel could improve reproducibility, clarify mechanistic phenomena, and enable human implantation of organoids (Hernandez-Gordillo et al., 2020). The application of organoids in regenerative medicine is considered to be very promising. For this purpose, changes in Matrigel are also essential. However, the organoids of some cancer patients not only grow slowly but also have a low success rate, especially in some advanced patients and cancers with a high degree of malignancy.

Some scientists have conducted related research regarding the mentioned problems about the maturation of organoids *in vitro*. It is demonstrated that uniaxial strain induces the growth and maturation of human intestinal organoids transplanted into mouse mesentery by compressed nitinol springs (Poling et al., 2018). Kwang Bo Jung et al. showed that coculture with human T lymphocytes could induce the *in vitro* maturation of human intestinal organoids and determined that interleukin 2 (IL-2) activates STAT3 as the main factor inducing maturation. Moreover, the matured human intestinal organoids *in vitro* still maintain their mature state after *in vivo* engraftment (Jung et al., 2018).

With the widespread application of organoids in life sciences, the standardization of organoids urgently needs to be resolved. First, the definition of organoids needs to be standardized. Some articles use “organoids,” but do not meet the definition of the organoid. The paper published in the *New England Journal of Medicine* gave a very classic definition, so that misuse should be avoided while using in the future. Notably, certain researchers have confused the concepts of tumor spheroids and organoids, who describe organoids in place of tumor spheroids in their studies.

Most of the published organoid culture operation processes need a conditioned medium. The composition of the medium may vary from batch to batch, while commercial culture can provide quality control of the culture medium and ensure stability between different batches. Therefore, try to use a commercial culture medium for organoid culture to improve repeatability and stability. There has always been a problem with experiments’ reproducibility within and between laboratories in the process of organoid culture. The difference in experimental methods, different manufacturers of experimental materials, and differences between batches are considered the main reason that affects experimental reproducibility. Hans Clevers pointed out that the field of organoids is highly dependent on complex experimental sequences. However, it is found that many studies refer to the first CRC organoid culture method that Hans Clevers wrote in 2011 by the literature survey on CRC organoid culture (Sato et al., 2011). On this basis, some minor adjustments have been made. Is it possible to conduct standardized research on CRC organoids based on this classic document?

Regarding the standardization of organoids, Lancaster said that “Sometimes standardization is important, but it can also be constraining on what you can learn if you cannot play around with your culture conditions.” Nature Research has updated the Protocols Exchange (https://protocolexchange.researchsquare.com/browse/journal/protocol-exchange), which provides a platform for researchers to exchange, discuss, and learn about the experimental procedures that are included in the publication. Many scientists believe that improving standardization is an important step to expand the clinical application of this technology. At present, organoids are mainly applied in personalized clinical medicine, and organoid culture is carried out for different patients to select the most suitable drug for treatment. Different patients’ organoid culture requires different conditions. Currently, standardization may limit the application of organoids in personalized medicine. We think the standardization of organoids should be the standardization of different situations, not a generalization. Although there is no consensus on the standardization of organoids, some results have been achieved. We and other peers are looking forward to solving these problems in future studies.

## Conclusion

In summary, CRC organoids provide excellent opportunities to study human CRC, breaking the limitations of the previous clinical and laboratory research and showing a broad application prospect. Human CRC organoids further our fundamental understanding of CRC occurrence and development, biology, and disease, promising tools for a wide range of biomedical applications, from disease modeling to drug screen and personalized medicine. Organoids are the bridge that connects clinical and scientific research by using organoids to study urgently needed problems in clinical cancer treatment and translating research results in clinical applications, which will better benefit human health. At present, CRC organoids derived from CRC patients were used for drug screening and guiding clinicians to individual treatment of patients, which has saved and could prolong the lives of CRC patients, especially the patients with advanced CRC. CRC has become an enormous factor threatening human health all over the world. In the future, organoids will still face many enormous challenges. The solution to these problems will require a multidisciplinary approach, so biologists, clinicians, and bioengineers need to work closely to explore many scientific problems of CRC further. The CRC organoid is used as an *in vitro* model. This model effectively combines the basic research and clinical treatment of CRC, breaking the original CRC treatment way, making the treatment of CRC patients enter the era of personalized medicine genuinely, and providing hope of life for more CRC patients. At present, the use of organoids derived from CRC patients for drug screening and guiding clinicians to individualized treatment of patients has saved the lives of some people, especially patients with advanced CRC.

## References

[B1] Abdul L. Rajasekar S. Lin D. S. Y. Venkatasubramania Raja S. Sotra A. Feng Y. (2020). Deep-lumen Assay - Human Lung Epithelial Spheroid Classification from Brightfield Images Using Deep Learning. Lab. Chip 20, 4623–4631. 10.1039/d0lc01010c 33151236

[B2] Alves Martins B. A. de Bulhões G. F. Cavalcanti I. N. Martins M. M. de Oliveira P. G. Martins A. M. A. (2019). Biomarkers in Colorectal Cancer: The Role of Translational Proteomics Research. Front. Oncol. 9, 1284. 10.3389/fonc.2019.01284 31828035 PMC6890575

[B3] Aono S. Hatanaka A. Hatanaka A. Gao Y. Hippo Y. Taketo M. M. (2019). β-Catenin/TCF4 Complex-Mediated Induction of the NRF3 (NFE2L3) Gene in Cancer Cells. Ijms 20, 3344. 10.3390/ijms20133344 PMC6651286 31288376

[B4] Armando R. Mengual Gomez D. Gomez D. (2020). New Drugs Are Not Enough-drug R-epositioning in O-ncology: An U-pdate. Int. J. Oncol. 56, 651–684. 10.3892/ijo.2020.4966 32124955 PMC7010222

[B5] Arrowsmith J. Miller P. (2013). Phase II and Phase III Attrition Rates 2011-2012. Nat. Rev. Drug Discov. 12, 569. 10.1038/nrd4090 23903212

[B6] Artegiani B. Hendriks D. Beumer J. Kok R. Zheng X. Joore I. (2020). Fast and Efficient Generation of Knock-In Human Organoids Using Homology-independent Crispr-Cas9 Precision Genome Editing. Nat. Cel Biol 22, 321–331. 10.1038/s41556-020-0472-5 32123335

[B7] Baptista P. M. Siddiqui M. M. Lozier G. Rodriguez S. R. Atala A. Soker S. (2011). The Use of Whole Organ Decellularization for the Generation of a Vascularized Liver Organoid. Hepatology 53, 604–617. 10.1002/hep.24067 21274881

[B8] Barker N. van Es J. H. Kuipers J. Kujala P. van den Born M. Cozijnsen M. (2007). Identification of Stem Cells in Small Intestine and colon by Marker Gene Lgr5. Nature 449, 1003–1007. 10.1038/nature06196 17934449

[B9] Ben-David U. Ha G. Tseng Y.-Y. Greenwald N. F. Oh C. Shih J. (2017). Patient-derived Xenografts Undergo Mouse-specific Tumor Evolution. Nat. Genet. 49, 1567–1575. 10.1038/ng.3967 28991255 PMC5659952

[B10] Ben-David U. Siranosian B. Ha G. Tang H. Oren Y. Hinohara K. (2018). Genetic and Transcriptional Evolution Alters Cancer Cell Line Drug Response. Nature 560, 325–330. 10.1038/s41586-018-0409-3 30089904 PMC6522222

[B11] Berkers G. van Mourik P. Vonk A. M. Kruisselbrink E. Dekkers J. F. de Winter-de Groot K. M. (2019). Rectal Organoids Enable Personalized Treatment of Cystic Fibrosis. Cel Rep. 26, 1701–1708. e1703. 10.1016/j.celrep.2019.01.068 30759382

[B12] Beshiri M. L. Tice C. M. Tran C. Nguyen H. M. Sowalsky A. G. Agarwal S. (2018). A Pdx/organoid Biobank of Advanced Prostate Cancers Captures Genomic and Phenotypic Heterogeneity for Disease Modeling and Therapeutic Screening. Clin. Cancer Res. 24, 4332–4345. 10.1158/1078-0432.ccr-18-0409 29748182 PMC6125202

[B13] Bolhaqueiro A. C. F. Ponsioen B. Bakker B. Klaasen S. J. Kucukkose E. van Jaarsveld R. H. (2019). Ongoing Chromosomal Instability and Karyotype Evolution in Human Colorectal Cancer Organoids. Nat. Genet. 51, 824–834. 10.1038/s41588-019-0399-6 31036964

[B14] Bradley C. A. (2018). Organoids Predict Clinical Responses. Nat. Rev. Gastroenterol. Hepatol. 15, 189. 10.1038/nrgastro.2018.25 29535457

[B15] Brandenberg N. Hoehnel S. Kuttler F. Homicsko K. Ceroni C. Ringel T. (2020). High-throughput Automated Organoid Culture via Stem-Cell Aggregation in Microcavity Arrays. Nat. Biomed. Eng. 4, 863–874. 10.1038/s41551-020-0565-2 32514094

[B16] Bray F. Ferlay J. Soerjomataram I. Siegel R. L. Torre L. A. Jemal A. (2018). Global Cancer Statistics 2018: Globocan Estimates of Incidence and Mortality Worldwide for 36 Cancers in 185 Countries. CA: A Cancer J. Clinicians 68, 394–424. 10.3322/caac.21492 30207593

[B17] Broutier L. Andersson-Rolf A. Hindley C. J. Boj S. F. Clevers H. Koo B.-K. (2016). Culture and Establishment of Self-Renewing Human and Mouse Adult Liver and Pancreas 3d Organoids and Their Genetic Manipulation. Nat. Protoc. 11, 1724–1743. 10.1038/nprot.2016.097 27560176

[B18] Bruun J. Kryeziu K. Eide P. W. Moosavi S. H. Eilertsen I. A. Langerud J. (2020). Patient-derived Organoids from Multiple Colorectal Cancer Liver Metastases Reveal Moderate Intra-patient Pharmacotranscriptomic Heterogeneity. Clin. Cancer Res. 26, 4107–4119. 10.1158/1078-0432.ccr-19-3637 32299813

[B19] Bu L. Baba H. Yoshida N. Miyake K. Yasuda T. Uchihara T. (2019). Biological Heterogeneity and Versatility of Cancer-Associated Fibroblasts in the Tumor Microenvironment. Oncogene 38, 4887–4901. 10.1038/s41388-019-0765-y 30816343

[B20] Buczacki S. J. A. Popova S. Biggs E. Koukorava C. Buzzelli J. Vermeulen L. (2018). Itraconazole Targets Cell Cycle Heterogeneity in Colorectal Cancer. J. Exp. Med. 215, 1891–1912. 10.1084/jem.20171385 29853607 PMC6028508

[B21] Buzzelli J. N. Ouaret D. Brown G. Allen P. D. Muschel R. J. (2018). Colorectal Cancer Liver Metastases Organoids Retain Characteristics of Original Tumor and Acquire Chemotherapy Resistance. Stem Cel Res. 27, 109–120. 10.1016/j.scr.2018.01.016 PMC5842239 29414601

[B22] Cakir B. Xiang Y. Tanaka Y. Kural M. H. Parent M. Kang Y.-J. (2019). Engineering of Human Brain Organoids with a Functional Vascular-like System. Nat. Methods 16, 1169–1175. 10.1038/s41592-019-0586-5 31591580 PMC6918722

[B23] Cao L. Kuratnik A. Xu W. Gibson J. D. Kolling F. Falcone E. R. (2015). Development of Intestinal Organoids as Tissue Surrogates: Cell Composition and the Epigenetic Control of Differentiation. Mol. Carcinog. 54, 189–202. 10.1002/mc.22089 24115167

[B24] Cattaneo C. M. Dijkstra K. K. Fanchi L. F. Kelderman S. Kaing S. van Rooij N. (2020). Tumor Organoid-T-Cell Coculture Systems. Nat. Protoc. 15, 15–39. 10.1038/s41596-019-0232-9 31853056 PMC7610702

[B25] Chen K.-Y. Srinivasan T. Lin C. Tung K.-L. Gao Z. Hsu D. S. (2018). Single-cell Transcriptomics Reveals Heterogeneity and Drug Response of Human Colorectal Cancer Organoids. Annu. Int. Conf. IEEE Eng. Med. Biol. Soc. 2018, 2378–2381. 10.1109/embc.2018.8512784 30440885 PMC6317967

[B26] Choi J.-I. Jang S. I. Hong J. Kim C. H. Kwon S. S. Park J. S. (2021). Cancer-initiating Cells in Human Pancreatic Cancer Organoids Are Maintained by Interactions with Endothelial Cells. Cancer Lett. 498, 42–53. 10.1016/j.canlet.2020.10.012 33188841

[B27] Clevers H. (2016). Modeling Development and Disease with Organoids. Cell 165, 1586–1597. 10.1016/j.cell.2016.05.082 27315476

[B28] Cong Y. Han X. Wang Y. Chen Z. Lu Y. Liu T. (2020). Drug Toxicity Evaluation Based on Organ-On-A-Chip Technology: A Review. Micromachines 11, 381. 10.3390/mi11040381 PMC7230535 32260191

[B29] Cook D. Brown D. Alexander R. March R. Morgan P. Satterthwaite G. (2014). Lessons Learned from the Fate of Astrazeneca's Drug Pipeline: A Five-Dimensional Framework. Nat. Rev. Drug Discov. 13, 419–431. 10.1038/nrd4309 24833294

[B30] Costales-Carrera A. Fernández-Barral A. Bustamante-Madrid P. Domínguez O. Guerra-Pastrián L. Cantero R. (2020). Comparative Study of Organoids from Patient-Derived normal and Tumor colon and Rectal Tissue. Cancers 12, 2302. 10.3390/cancers12082302 PMC7465167 32824266

[B31] Costales-Carrera A. Fernández-Barral A. Bustamante-Madrid P. Guerra L. Cantero R. Barbáchano A. (2019). Plocabulin Displays strong Cytotoxic Activity in a Personalized colon Cancer Patient-Derived 3d Organoid Assay. Mar. Drugs 17, 648. 10.3390/md17110648 PMC6891270 31752287

[B32] Dekkers J. F. Berkers G. Kruisselbrink E. Vonk A. de Jonge H. R. Janssens H. M. (2016). Characterizing Responses to Cftr-Modulating Drugs Using Rectal Organoids Derived from Subjects with Cystic Fibrosis. Sci. Transl. Med. 8, 344ra384. 10.1126/scitranslmed.aad8278 27334259

[B33] Dekkers J. F. Whittle J. R. Vaillant F. Chen H.-R. Dawson C. Liu K. (2020). Modeling Breast Cancer Using Crispr-Cas9-Mediated Engineering of Human Breast Organoids. J. Natl. Cancer Inst. 112, 540–544. 10.1093/jnci/djz196 31589320 PMC7225674

[B34] Dekkers J. F. Wiegerinck C. L. de Jonge H. R. Bronsveld I. Janssens H. M. de Winter-de Groot K. M. (2013). A Functional Cftr Assay Using Primary Cystic Fibrosis Intestinal Organoids. Nat. Med. 19, 939–945. 10.1038/nm.3201 23727931

[B35] Dijkstra K. K. Cattaneo C. M. Weeber F. Chalabi M. van de Haar J. Fanchi L. F. (2018). Generation of Tumor-Reactive T Cells by Co-culture of Peripheral Blood Lymphocytes and Tumor Organoids. Cell 174, 1586–1598. e1512. 10.1016/j.cell.2018.07.009 30100188 PMC6558289

[B36] Driehuis E. van Hoeck A. Moore K. Kolders S. Francies H. E. Gulersonmez M. C. (2019). Pancreatic Cancer Organoids Recapitulate Disease and Allow Personalized Drug Screening. Proc. Natl. Acad. Sci. USA 116, 26580–26590. 10.1073/pnas.1911273116 PMC6936689 31818951

[B37] Drost J. van Jaarsveld R. H. Ponsioen B. Zimberlin C. van Boxtel R. Buijs A. (2015). Sequential Cancer Mutations in Cultured Human Intestinal Stem Cells. Nature 521, 43–47. 10.1038/nature14415 25924068

[B38] Du G.-W. Yan X. Chen Z. Zhang R.-J. Tuoheti K. Bai X.-J. (2020). Identification of Transforming Growth Factor Beta Induced (Tgfbi) as an Immune-Related Prognostic Factor in clear Cell Renal Cell Carcinoma (Ccrcc). Aging 12, 8484–8505. 10.18632/aging.103153 32406866 PMC7244045

[B39] Dzutsev A. Badger J. H. Perez-Chanona E. Roy S. Salcedo R. Smith C. K. (2017). Microbes and Cancer. Annu. Rev. Immunol. 35, 199–228. 10.1146/annurev-immunol-051116-052133 28142322

[B40] Eder A. Vollert I. Hansen A. Eschenhagen T. (2016). Human Engineered Heart Tissue as a Model System for Drug Testing. Adv. Drug Deliv. Rev. 96, 214–224. 10.1016/j.addr.2015.05.010 26026976

[B41] Fang G. Lu H. Al-Nakashli R. Chapman R. Zhang Y. Ju L. A. (2021). Enabling Peristalsis of Human colon Tumor Organoids on Microfluidic Chips. Biofabrication 14, 015006. 10.1088/1758-5090/ac2ef9 34638112

[B42] Fatehullah A. Tan S. H. Barker N. (2016). Organoids as an *In Vitro* Model of Human Development and Disease. Nat. Cel Biol 18, 246–254. 10.1038/ncb3312 26911908

[B43] Fearon E. R. (2011). Molecular Genetics of Colorectal Cancer. Annu. Rev. Pathol. Mech. Dis. 6, 479–507. 10.1146/annurev-pathol-011110-130235 21090969

[B44] Fearon E. R. Vogelstein B. (1990). A Genetic Model for Colorectal Tumorigenesis. Cell 61, 759–767. 10.1016/0092-8674(90)90186-i 2188735

[B45] Fessler E. Drost J. Hooff S. R. Linnekamp J. F. Wang X. Jansen M. (2016). TGFβ Signaling Directs Serrated Adenomas to the Mesenchymal Colorectal Cancer Subtype. EMBO Mol. Med. 8, 745–760. 10.15252/emmm.201606184 27221051 PMC4931289

[B46] Fujii M. Matano M. Nanki K. Sato T. (2015). Efficient Genetic Engineering of Human Intestinal Organoids Using Electroporation. Nat. Protoc. 10, 1474–1485. 10.1038/nprot.2015.088 26334867

[B47] Fujii M. Matano M. Toshimitsu K. Takano A. Mikami Y. Nishikori S. (2018). Human Intestinal Organoids Maintain Self-Renewal Capacity and Cellular Diversity in Niche-Inspired Culture Condition. Cell Stem Cell 23, 787–793. e786. 10.1016/j.stem.2018.11.016 30526881

[B48] Fujii M. Shimokawa M. Date S. Takano A. Matano M. Nanki K. (2016). A Colorectal Tumor Organoid Library Demonstrates Progressive Loss of Niche Factor Requirements during Tumorigenesis. Cell Stem Cell 18, 827–838. 10.1016/j.stem.2016.04.003 27212702

[B49] Fujii M. Sugimoto S. Sato T. (2020). Linking Human Intestinal Scaffolds and Organoids to Combat Intestinal Failure. Nat. Med. 26, 1517–1518. 10.1038/s41591-020-1096-9 32968235

[B50] Fumagalli A. Drost J. Suijkerbuijk S. J. E. van Boxtel R. de Ligt J. Offerhaus G. J. (2017). Genetic Dissection of Colorectal Cancer Progression by Orthotopic Transplantation of Engineered Cancer Organoids. Proc. Natl. Acad. Sci. USA 114, E2357–e2364. 10.1073/pnas.1701219114 28270604 PMC5373343

[B51] Fumagalli A. Suijkerbuijk S. J. E. Begthel H. Beerling E. Oost K. C. Snippert H. J. (2018). A Surgical Orthotopic Organoid Transplantation Approach in Mice to Visualize and Study Colorectal Cancer Progression. Nat. Protoc. 13, 235–247. 10.1038/nprot.2017.137 29300390

[B52] Ganesh K. Wu C. O’Rourke K. P. Szeglin B. C. Zheng Y. Sauvé C.-E. G. (2019). A Rectal Cancer Organoid Platform to Study Individual Responses to Chemoradiation. Nat. Med. 25, 1607–1614. 10.1038/s41591-019-0584-2 31591597 PMC7385919

[B53] Ganzleben I. Hohmann M. Grünberg A. Gonzales-Menezes J. Vieth M. Liebing E. (2020). Topical application of chlorin e6-pvp (ce6-pvp) for improved endoscopic detection of neoplastic lesions in a murine colitis-associated cancer model. Sci. Rep. 10, 13129. 10.1038/s41598-020-69570-2 32753653 PMC7403373

[B54] Gao D. Vela I. Sboner A. Iaquinta P. J. Karthaus W. R. Gopalan A. (2014). Organoid Cultures Derived from Patients with Advanced Prostate Cancer. Cell 159, 176–187. 10.1016/j.cell.2014.08.016 25201530 PMC4237931

[B55] Goto N. Fukuda A. Yamaga Y. Yoshikawa T. Maruno T. Maekawa H. (2019). Lineage Tracing and Targeting of IL17RB+tuft Cell-like Human Colorectal Cancer Stem Cells. Proc. Natl. Acad. Sci. USA 116, 12996–13005. 10.1073/pnas.1900251116 31182574 PMC6601016

[B56] Guinney J. Dienstmann R. Wang X. de Reyniès A. Schlicker A. Soneson C. (2015). The Consensus Molecular Subtypes of Colorectal Cancer. Nat. Med. 21, 1350–1356. 10.1038/nm.3967 26457759 PMC4636487

[B57] Ham O. Jin Y. B. Kim J. Lee M.-O. (2020). Blood Vessel Formation in Cerebral Organoids Formed from Human Embryonic Stem Cells. Biochem. Biophysical Res. Commun. 521, 84–90. 10.1016/j.bbrc.2019.10.079 31629471

[B58] Han M. Cheng X. Gao Z. Zhao R. Zhang S. (2017). Inhibition of Tumor Cell Growth by Adenine Is Mediated by Apoptosis Induction and Cell Cycle S Phase Arrest. Oncotarget 8, 94286–94296. 10.18632/oncotarget.21690 29212228 PMC5706874

[B59] He C. Ruan F. Jiang S. Zeng J. Yin H. Liu R. (2020). Black Phosphorus Quantum Dots Cause Nephrotoxicity in Organoids, Mice, and Human Cells. Small 16, 2001371. 10.1002/smll.202001371 32338439

[B60] Hernandez-Gordillo V. Kassis T. Lampejo A. Choi G. Gamboa M. E. Gnecco J. S. (2020). Fully Synthetic Matrices for *In Vitro* Culture of Primary Human Intestinal Enteroids and Endometrial Organoids. Biomaterials 254, 120125. 10.1016/j.biomaterials.2020.120125 32502894 PMC8005336

[B61] Hill S. J. Decker B. Roberts E. A. Horowitz N. S. Muto M. G. Worley M. J. Jr. (2018). Prediction of DNA Repair Inhibitor Response in Short-Term Patient-Derived Ovarian Cancer Organoids. Cancer Discov. 8, 1404–1421. 10.1158/2159-8290.cd-18-0474 30213835 PMC6365285

[B62] Hoang P. Wang J. Conklin B. R. Healy K. E. Ma Z. (2018). Generation of Spatial-Patterned Early-Developing Cardiac Organoids Using Human Pluripotent Stem Cells. Nat. Protoc. 13, 723–737. 10.1038/nprot.2018.006 29543795 PMC6287283

[B63] Holloway E. M. Wu J. H. Czerwinski M. Sweet C. W. Wu A. Tsai Y.-H. (2020). Differentiation of Human Intestinal Organoids with Endogenous Vascular Endothelial Cells. Developmental Cel 54, 516–528. e517. 10.1016/j.devcel.2020.07.023 PMC7480827 32841595

[B64] Hu C. Chen M. Jiang R. Guo Y. Wu M. Zhang X. (2018). Exosome-related Tumor Microenvironment. J. Cancer 9, 3084–3092. 10.7150/jca.26422 30210631 PMC6134819

[B65] Hu X. Zhang L. Li Y. Ma X. Dai W. Gao X. (2020). Organoid Modelling Identifies that Dach1 Functions as a Tumour Promoter in Colorectal Cancer by Modulating Bmp Signalling. EBioMedicine 56, 102800. 10.1016/j.ebiom.2020.102800 32512510 PMC7281795

[B66] Hu Y. Sui X. Song F. Li Y. Li K. Chen Z. (2021). Lung Cancer Organoids Analyzed on Microwell Arrays Predict Drug Responses of Patients within a Week. Nat. Commun. 12, 2581. 10.1038/s41467-021-22676-1 33972544 PMC8110811

[B67] Huch M. Gehart H. van Boxtel R. Hamer K. Blokzijl F. Verstegen M. M. A. (2015). Long-term Culture of Genome-Stable Bipotent Stem Cells from Adult Human Liver. Cell 160, 299–312. 10.1016/j.cell.2014.11.050 25533785 PMC4313365

[B68] Iida N. Dzutsev A. Stewart C. A. Smith L. Bouladoux N. Weingarten R. A. (2013). Commensal Bacteria Control Cancer Response to Therapy by Modulating the Tumor Microenvironment. Science 342, 967–970. 10.1126/science.1240527 24264989 PMC6709532

[B69] Imamura Y. Mukohara T. Shimono Y. Funakoshi Y. Chayahara N. Toyoda M. (2015). Comparison of 2d- and 3d-Culture Models as Drug-Testing Platforms in Breast Cancer. Oncol. Rep. 33, 1837–1843. 10.3892/or.2015.3767 25634491

[B70] Jacob F. Salinas R. D. Zhang D. Y. Nguyen P. T. T. Schnoll J. G. Wong S. Z. H. (2020). A Patient-Derived Glioblastoma Organoid Model and Biobank Recapitulates Inter- and Intra-tumoral Heterogeneity. Cell 180, 188–204. e122. 10.1016/j.cell.2019.11.036 31883794 PMC7556703

[B71] Jamal S. Ali W. Nagpal P. Grover S. Grover A. (2019). Computational Models for the Prediction of Adverse Cardiovascular Drug Reactions. J. Transl Med. 17, 171. 10.1186/s12967-019-1918-z 31118067 PMC6530172

[B72] Janakiraman H. Zhu Y. Becker S. A. Wang C. Cross A. Curl E. (2020). Modeling Rectal Cancer to advance Neoadjuvant Precision Therapy. Int. J. Cancer 147, 1405–1418. 10.1002/ijc.32876 31989583

[B73] Jardé T. Chan W. H. Rossello F. J. Kaur Kahlon T. Theocharous M. Kurian Arackal T. (2020). Mesenchymal Niche-Derived Neuregulin-1 Drives Intestinal Stem Cell Proliferation and Regeneration of Damaged Epithelium. Cell Stem Cell 27, 646–662. e647. 10.1016/j.stem.2020.06.021 32693086

[B74] Jiang S. Zhao H. Zhang W. Wang J. Liu Y. Cao Y. (2020). An Automated Organoid Platform with Inter-organoid Homogeneity and Inter-patient Heterogeneity. Cel Rep. Med. 1, 100161. 10.1016/j.xcrm.2020.100161 PMC7762778 33377132

[B75] Jiang W. Xie S. Liu Y. Zou S. Zhu X. (2020). The Application of Patient-Derived Xenograft Models in Gynecologic Cancers. J. Cancer 11, 5478–5489. 10.7150/jca.46145 32742495 PMC7391187

[B76] Joshi P. Datar A. Yu K.-N. Kang S.-Y. Lee M.-Y. (2018). High-content Imaging Assays on a Miniaturized 3d Cell Culture Platform. Toxicol. Vitro 50, 147–159. 10.1016/j.tiv.2018.02.014 PMC5994365 29501531

[B77] Jung E. Choi J. Kim J.-S. Han T.-S. (2021). Microrna-based Therapeutics for Drug-Resistant Colorectal Cancer. Pharmaceuticals 14, 136. 10.3390/ph14020136 33567635 PMC7915952

[B78] Jung K. B. Lee H. Son Y. S. Lee M.-O. Kim Y.-D. Oh S. J. (2018). Interleukin-2 Induces the *In Vitro* Maturation of Human Pluripotent Stem Cell-Derived Intestinal Organoids. Nat. Commun. 9, 3039. 10.1038/s41467-018-05450-8 30072687 PMC6072745

[B79] Junttila M. R. de Sauvage F. J. (2013). Influence of Tumour Micro-environment Heterogeneity on Therapeutic Response. Nature 501, 346–354. 10.1038/nature12626 24048067

[B80] Karthaus W. R. Iaquinta P. J. Drost J. Gracanin A. van Boxtel R. Wongvipat J. (2014). Identification of Multipotent Luminal Progenitor Cells in Human Prostate Organoid Cultures. Cell 159, 163–175. 10.1016/j.cell.2014.08.017 25201529 PMC4772677

[B81] Keum N. Giovannucci E. (2019). Global burden of Colorectal Cancer: Emerging Trends, Risk Factors and Prevention Strategies. Nat. Rev. Gastroenterol. Hepatol. 16, 713–732. 10.1038/s41575-019-0189-8 31455888

[B82] Kim M. Mun H. Sung C. O. Cho E. J. Jeon H.-J. Chun S.-M. (2019). Patient-derived Lung Cancer Organoids as *In Vitro* Cancer Models for Therapeutic Screening. Nat. Commun. 10, 3991. 10.1038/s41467-019-11867-6 31488816 PMC6728380

[B83] Knight J. R. P. Alexandrou C. Skalka G. L. Vlahov N. Pennel K. Officer L. (2021). Mnk inhibition sensitizes kras-mutant colorectal cancer to mtorc1 inhibition by reducing eif4e phosphorylation and c-myc expression. Cancer Discov. 11, 1228–1247. 10.1158/2159-8290.cd-20-0652 33328217 PMC7611341

[B84] Koike H. Iwasawa K. Ouchi R. Maezawa M. Giesbrecht K. Saiki N. (2019). Modelling Human Hepato-Biliary-Pancreatic Organogenesis from the Foregut-Midgut Boundary. Nature 574, 112–116. 10.1038/s41586-019-1598-0 31554966 PMC7643931

[B85] Kramer C. D. Genco C. A. (2017). Microbiota, Immune Subversion, and Chronic Inflammation. Front. Immunol. 8, 255. 10.3389/fimmu.2017.00255 28348558 PMC5346547

[B86] Kuipers E. J. Grady W. M. Lieberman D. Seufferlein T. Sung J. J. Boelens P. G. (2015). Colorectal Cancer. Nat. Rev. Dis. Primers 1, 15065. 10.1038/nrdp.2015.65 27189416 PMC4874655

[B87] Kupfer M. E. Lin W.-H. Ravikumar V. Qiu K. Wang L. Gao L. (2020). *In Situ* expansion, Differentiation, and Electromechanical Coupling of Human Cardiac Muscle in a 3d Bioprinted, Chambered Organoid. Circ. Res. 127, 207–224. 10.1161/circresaha.119.316155 32228120 PMC8210857

[B88] Lamers M. M. Beumer J. van der Vaart J. Knoops K. Puschhof J. Breugem T. I. (2020). Sars-cov-2 Productively Infects Human Gut Enterocytes. Science 369, 50–54. 10.1126/science.abc1669 32358202 PMC7199907

[B89] Lancaster M. A. Huch M. (2019). Disease Modelling in Human Organoids. Dis. Model. Mech. 12, dmm039347. 10.1242/dmm.039347 31383635 PMC6679380

[B90] Lancaster M. A. Renner M. Martin C.-A. Wenzel D. Bicknell L. S. Hurles M. E. (2013). Cerebral Organoids Model Human Brain Development and Microcephaly. Nature 501, 373–379. 10.1038/nature12517 23995685 PMC3817409

[B91] Lau H. C. H. Kranenburg O. Xiao H. Yu J. (2020). Organoid Models of Gastrointestinal Cancers in Basic and Translational Research. Nat. Rev. Gastroenterol. Hepatol. 17, 203–222. 10.1038/s41575-019-0255-2 32099092

[B92] Lee S. H. Hu W. Matulay J. T. Silva M. V. Owczarek T. B. Kim K. (2018). Tumor Evolution and Drug Response in Patient-Derived Organoid Models of Bladder Cancer. Cell 173, 515–528. e517. 10.1016/j.cell.2018.03.017 29625057 PMC5890941

[B93] Lee Y.-J. Cho J.-M. Sai S. Oh J. Y. Park J.-A. Oh S. J. (2019). 5-fluorouracil as a Tumor-Treating Field-Sensitizer in colon Cancer Therapy. Cancers 11, 1999. 10.3390/cancers11121999 PMC6966590 31842288

[B94] Li H. Dai W. Xia X. Wang R. Zhao J. Han L. (2020). Modeling Tumor Development and Metastasis Using Paired Organoids Derived from Patients with Colorectal Cancer Liver Metastases. J. Hematol. Oncol. 13, 119. 10.1186/s13045-020-00957-4 32883331 PMC7650218

[B95] Li M. Izpisua Belmonte J. C. (2019). Organoids - Preclinical Models of Human Disease. N. Engl. J. Med. 380, 569–579. 10.1056/NEJMra1806175 30726695

[B96] Liang X. Xie R. Su J. Ye B. Wei S. Liang Z. (2019). Inhibition of Rna Polymerase Iii Transcription by Triptolide Attenuates Colorectal Tumorigenesis. J. Exp. Clin. Cancer Res. 38, 217. 10.1186/s13046-019-1232-x 31122284 PMC6533717

[B97] Lindemans C. A. Calafiore M. Mertelsmann A. M. O’Connor M. H. Dudakov J. A. Jenq R. R. (2015). Interleukin-22 Promotes Intestinal-Stem-Cell-Mediated Epithelial Regeneration. Nature 528, 560–564. 10.1038/nature16460 26649819 PMC4720437

[B98] Liu Y. Deguchi Y. Tian R. Wei D. Wu L. Chen W. (2019). Pleiotropic Effects of Ppard Accelerate Colorectal Tumorigenesis, Progression, and Invasion. Cancer Res. 79, 954–969. 10.1158/0008-5472.can-18-1790 30679176 PMC6397663

[B99] Lorenzi F. Babaei-Jadidi R. Sheard J. Spencer-Dene B. Nateri A. S. (2016). Fbxw7-associated Drug Resistance Is Reversed by Induction of Terminal Differentiation in Murine Intestinal Organoid Culture. Mol. Ther. - Methods Clin. Development 3, 16024. 10.1038/mtm.2016.24 PMC4830362 27110583

[B100] Low J. H. Li P. Chew E. G. Y. Zhou B. Suzuki K. Zhang T. (2019). Generation of Human Psc-Derived Kidney Organoids with Patterned Nephron Segments and a De Novo Vascular Network. Cell Stem Cell 25, 373–387. e379. 10.1016/j.stem.2019.06.009 31303547 PMC6731150

[B101] Mager L. F. Burkhard R. Pett N. Cooke N. C. A. Brown K. Ramay H. (2020). Microbiome-derived Inosine Modulates Response to Checkpoint Inhibitor Immunotherapy. Science 369, 1481–1489. 10.1126/science.abc3421 32792462

[B102] Mallone A. Hosseini V. Chahbi K. Haenseler W. Vogel V. Weber B. (2021). Human Induced Pluripotent Stem Cell-Derived Vessels as Dynamic Atherosclerosis Model on a Chip.

[B103] Mansour A. A. Gonçalves J. T. Bloyd C. W. Li H. Fernandes S. Quang D. (2018). An *In Vivo* Model of Functional and Vascularized Human Brain Organoids. Nat. Biotechnol. 36, 432–441. 10.1038/nbt.4127 29658944 PMC6331203

[B104] Matano M. Date S. Shimokawa M. Takano A. Fujii M. Ohta Y. (2015). Modeling Colorectal Cancer Using Crispr-Cas9-Mediated Engineering of Human Intestinal Organoids. Nat. Med. 21, 256–262. 10.1038/nm.3802 25706875

[B105] McMillin D. W. Delmore J. Weisberg E. Negri J. M. Geer D. C. Klippel S. (2010). Tumor Cell-specific Bioluminescence Platform to Identify Stroma-Induced Changes to Anticancer Drug Activity. Nat. Med. 16, 483–489. 10.1038/nm.2112 20228816 PMC3786785

[B106] Mekky G. Seeds M. Diab A. E. A. A. Shehata A. M. Ahmed‐Farid O. A. H. Alzebdeh D. (2021). The Potential Toxic Effects of Magnesium Oxide Nanoparticles and Valproate on Liver Tissue. J. Biochem. Mol. Toxicol. 35, e22676. 10.1002/jbt.22676 33315275

[B107] Meric-Bernstam F. Brusco L. Shaw K. Horombe C. Kopetz S. Davies M. A. (2015). Feasibility of Large-Scale Genomic Testing to Facilitate Enrollment onto Genomically Matched Clinical Trials. Jco 33, 2753–2762. 10.1200/jco.2014.60.4165 PMC4550690 26014291

[B108] Mittal R. Woo F. W. Castro C. S. Cohen M. A. Karanxha J. Mittal J. (2019). Organ‐on‐chip Models: Implications in Drug Discovery and Clinical Applications. J. Cel Physiol 234, 8352–8380. 10.1002/jcp.27729 30443904

[B109] Moran O. (2017). The Gating of the Cftr Channel. Cell. Mol. Life Sci. 74, 85–92. 10.1007/s00018-016-2390-z 27696113 PMC11107742

[B110] Morizane R. Lam A. Q. Freedman B. S. Kishi S. Valerius M. T. Bonventre J. V. (2015). Nephron Organoids Derived from Human Pluripotent Stem Cells Model Kidney Development and Injury. Nat. Biotechnol. 33, 1193–1200. 10.1038/nbt.3392 26458176 PMC4747858

[B111] Mosa M. H. Michels B. E. Menche C. Nicolas A. M. Darvishi T. Greten F. R. (2020). A Wnt-Induced Phenotypic Switch in Cancer-Associated Fibroblasts Inhibits Emt in Colorectal Cancer. Cancer Res. 80, 5569–5582. 10.1158/0008-5472.can-20-0263 33055221

[B112] Mukohyama J. Isobe T. Hu Q. Hayashi T. Watanabe T. Maeda M. (2019). Mir-221 Targets Qki to Enhance the Tumorigenic Capacity of Human Colorectal Cancer Stem Cells. Cancer Res. 79, 5151–5158. 10.1158/0008-5472.can-18-3544 31416845 PMC6801097

[B113] Mullenders J. de Jongh E. Brousali A. Roosen M. Blom J. P. A. Begthel H. (2019). Mouse and Human Urothelial Cancer Organoids: A Tool for Bladder Cancer Research. Proc. Natl. Acad. Sci. USA 116, 4567–4574. 10.1073/pnas.1803595116 30787188 PMC6410883

[B114] Mun S. J. Ryu J.-S. Lee M.-O. Son Y. S. Oh S. J. Cho H.-S. (2019). Generation of Expandable Human Pluripotent Stem Cell-Derived Hepatocyte-like Liver Organoids. J. Hepatol. 71, 970–985. 10.1016/j.jhep.2019.06.030 31299272

[B115] Narasimhan V. Wright J. A. Churchill M. Wang T. Rosati R. Lannagan T. R. M. (2020). Medium-throughput Drug Screening of Patient-Derived Organoids from Colorectal Peritoneal Metastases to Direct Personalized Therapy. Clin. Cancer Res. 26, 3662–3670. 10.1158/1078-0432.ccr-20-0073 32376656 PMC8366292

[B116] Neal J. T. Li X. Zhu J. Giangarra V. Grzeskowiak C. L. Ju J. (2018). Organoid Modeling of the Tumor Immune Microenvironment. Cell 175, 1972–1988. e1916. 10.1016/j.cell.2018.11.021 30550791 PMC6656687

[B117] Ng S. Tan W. J. Pek M. M. X. Tan M.-H. Kurisawa M. (2019). Mechanically and Chemically Defined Hydrogel Matrices for Patient-Derived Colorectal Tumor Organoid Culture. Biomaterials 219, 119400. 10.1016/j.biomaterials.2019.119400 31398570

[B118] Nguyen A. T. Q. Lee S. y. Chin H. J. Le Q. V. C. Lee D. (2020). Kinase Activity of Erbb3 Contributes to Intestinal Organoids Growth and Intestinal Tumorigenesis. Cancer Sci. 111, 137–147. 10.1111/cas.14235 31724799 PMC6942447

[B119] Nguyen L. H. Liu P.-H. Zheng X. Keum N. Zong X. Li X. (2018). Sedentary Behaviors, Tv Viewing Time, and Risk of Young-Onset Colorectal Cancer. JNCI Cancer Spectr. 2, pky073. 10.1093/jncics/pky073 30740587 PMC6361621

[B120] Nikolić M. Z. Caritg O. Jeng Q. Johnson J.-A. Sun D. Howell K. J. (2017). Human Embryonic Lung Epithelial Tips Are Multipotent Progenitors that Can Be Expanded *In Vitro* as Long-Term Self-Renewing Organoids. Elife 6, e26575. 10.7554/eLife.26575 28665271 PMC5555721

[B121] O'Rourke K. P. Loizou E. Livshits G. Schatoff E. M. Baslan T. Manchado E. (2017). Transplantation of Engineered Organoids Enables Rapid Generation of Metastatic Mouse Models of Colorectal Cancer. Nat. Biotechnol. 35, 577–582. 10.1038/nbt.3837 28459450 PMC5462850

[B122] Ooft S. N. Weeber F. Dijkstra K. K. McLean C. M. Kaing S. van Werkhoven E. (2019). Patient-derived Organoids Can Predict Response to Chemotherapy in Metastatic Colorectal Cancer Patients. Sci. Transl. Med. 11, eaay2574. 10.1126/scitranslmed.aay2574 31597751

[B123] Oszvald Á. Szvicsek Z. Pápai M. Kelemen A. Varga Z. Tölgyes T. (2020a). Fibroblast-derived Extracellular Vesicles Induce Colorectal Cancer Progression by Transmitting Amphiregulin. Front. Cel Dev. Biol. 8, 558. 10.3389/fcell.2020.00558 PMC7381355 32775326

[B124] Oszvald Á. Szvicsek Z. Sándor G. O. Kelemen A. Soós A. Á. Pálóczi K. (2020b). Extracellular Vesicles Transmit Epithelial Growth Factor Activity in the Intestinal Stem Cell Niche. Stem Cells 38, 291–300. 10.1002/stem.3113 31675158

[B125] Otte J. Dizdar L. Behrens B. Goering W. Knoefel W. T. Wruck W. (2019). Fgf Signalling in the Self-Renewal of colon Cancer Organoids. Sci. Rep. 9, 17365. 10.1038/s41598-019-53907-7 31758153 PMC6874569

[B126] Palikuqi B. Nguyen D.-H. T. Li G. Schreiner R. Pellegata A. F. Liu Y. (2020). Adaptable Haemodynamic Endothelial Cells for Organogenesis and Tumorigenesis. Nature 585, 426–432. 10.1038/s41586-020-2712-z 32908310 PMC7480005

[B127] Park M. Kwon J. Shin H. J. Moon S. Kim S. Shin U. (2020). Butyrate Enhances the Efficacy of Radiotherapy via FOXO3A in Colorectal Cancer Patient-derived O-rganoids. Int. J. Oncol. 57, 1307–1318. 10.3892/ijo.2020.5132 33173975 PMC7646587

[B128] Park S. E. Georgescu A. Huh D. (2019). Organoids-on-a-chip. Science 364, 960–965. 10.1126/science.aaw7894 31171693 PMC7764943

[B129] Pasch C. A. Favreau P. F. Yueh A. E. Babiarz C. P. Gillette A. A. Sharick J. T. (2019). Patient-derived Cancer Organoid Cultures to Predict Sensitivity to Chemotherapy and Radiation. Clin. Cancer Res. 25, 5376–5387. 10.1158/1078-0432.ccr-18-3590 31175091 PMC6726566

[B130] Pauli C. Hopkins B. D. Prandi D. Shaw R. Fedrizzi T. Sboner A. (2017). Personalized *In Vitro* and *In Vivo* Cancer Models to Guide Precision Medicine. Cancer Discov. 7, 462–477. 10.1158/2159-8290.cd-16-1154 28331002 PMC5413423

[B131] Peel S. Corrigan A. M. Ehrhardt B. Jang K.-J. Caetano-Pinto P. Boeckeler M. (2019). Introducing an Automated High Content Confocal Imaging Approach for Organs-On-Chips. Lab. Chip 19, 410–421. 10.1039/c8lc00829a 30663729

[B132] Pham M. T. Pollock K. M. Rose M. D. Cary W. A. Stewart H. R. Zhou P. (2018). Generation of Human Vascularized Brain Organoids. Neuroreport 29, 588–593. 10.1097/wnr.0000000000001014 29570159 PMC6476536

[B133] Pillaiyar T. Meenakshisundaram S. Manickam M. Sankaranarayanan M. (2020). A Medicinal Chemistry Perspective of Drug Repositioning: Recent Advances and Challenges in Drug Discovery. Eur. J. Med. Chem. 195, 112275. 10.1016/j.ejmech.2020.112275 32283298 PMC7156148

[B134] Pleguezuelos-Manzano C. Puschhof J. Rosendahl Huber A. van Hoeck A. Wood H. M. Nomburg J. (2020). Mutational Signature in Colorectal Cancer Caused by Genotoxic Pks + *E. coli* . Nature 580, 269–273. 10.1038/s41586-020-2080-8 32106218 PMC8142898

[B135] Poling H. M. Wu D. Brown N. Baker M. Hausfeld T. A. Huynh N. (2018). Mechanically Induced Development and Maturation of Human Intestinal Organoids *In Vivo* . Nat. Biomed. Eng. 2, 429–442. 10.1038/s41551-018-0243-9 30151330 PMC6108544

[B136] Puca L. Bareja R. Prandi D. Shaw R. Benelli M. Karthaus W. R. (2018). Patient Derived Organoids to Model Rare Prostate Cancer Phenotypes. Nat. Commun. 9, 2404. 10.1038/s41467-018-04495-z 29921838 PMC6008438

[B137] Rajasekar S. Lin D. S. Y. Abdul L. Liu A. Sotra A. Zhang F. (2020). IFlowPlate-A Customized 384‐Well Plate for the Culture of Perfusable Vascularized Colon Organoids. Adv. Mater. 32, 2002974. 10.1002/adma.202002974 33000879

[B138] Reischmann N. Andrieux G. Griffin R. Reinheckel T. Boerries M. Brummer T. (2020). BRAFV600E Drives Dedifferentiation in Small Intestinal and Colonic Organoids and Cooperates with Mutant P53 and Apc Loss in Transformation. Oncogene 39, 6053–6070. 10.1038/s41388-020-01414-9 32792685 PMC7498370

[B139] Relier S. Yazdani L. Ayad O. Choquet A. Bourgaux J.-F. Prudhomme M. (2016). Antibiotics Inhibit Sphere-Forming Ability in Suspension Culture. Cancer Cel Int 16, 6. 10.1186/s12935-016-0277-6 PMC4751670 26877710

[B140] Rennert K. Steinborn S. Gröger M. Ungerböck B. Jank A.-M. Ehgartner J. (2015). A Microfluidically Perfused Three Dimensional Human Liver Model. Biomaterials 71, 119–131. 10.1016/j.biomaterials.2015.08.043 26322723

[B141] Richards D. J. Li Y. Kerr C. M. Yao J. Beeson G. C. Coyle R. C. (2020). Human Cardiac Organoids for the Modelling of Myocardial Infarction and Drug Cardiotoxicity. Nat. Biomed. Eng. 4, 446–462. 10.1038/s41551-020-0539-4 32284552 PMC7422941

[B142] Ringel T. Frey N. Ringnalda F. Janjuha S. Cherkaoui S. Butz S. (2020). Genome-Scale CRISPR Screening in Human Intestinal Organoids Identifies Drivers of TGF-β Resistance. Cell Stem Cell 26, 431–440. e438. 10.1016/j.stem.2020.02.007 32142663

[B143] Rocha F. G. Sundback C. A. Krebs N. J. Leach J. K. Mooney D. J. Ashley S. W. (2008). The Effect of Sustained Delivery of Vascular Endothelial Growth Factor on Angiogenesis in Tissue-Engineered Intestine. Biomaterials 29, 2884–2890. 10.1016/j.biomaterials.2008.03.026 18396329 PMC2685178

[B144] Rock J. R. Onaitis M. W. Rawlins E. L. Lu Y. Clark C. P. Xue Y. (2009). Basal Cells as Stem Cells of the Mouse Trachea and Human Airway Epithelium. Proc. Natl. Acad. Sci. 106, 12771–12775. 10.1073/pnas.0906850106 19625615 PMC2714281

[B145] Romero-López M. Trinh A. L. Sobrino A. Hatch M. M. S. Keating M. T. Fimbres C. (2017). Recapitulating the Human Tumor Microenvironment: Colon Tumor-Derived Extracellular Matrix Promotes Angiogenesis and Tumor Cell Growth. Biomaterials 116, 118–129. 10.1016/j.biomaterials.2016.11.034 27914984 PMC5226635

[B146] Roney M. S. I. Lanagan C. Sheng Y. H. Lawler K. Schmidt C. Nguyen N. T. (2021). IgM and IgA Augmented Autoantibody Signatures Improve Early‐stage Detection of Colorectal Cancer Prior to Nodal and Distant Spread. Clin. Transl Immunol. 10, e1330. 10.1002/cti2.1330 PMC8473921 34603722

[B147] Roper J. Tammela T. Cetinbas N. M. Akkad A. Roghanian A. Rickelt S. (2017). *In Vivo* genome Editing and Organoid Transplantation Models of Colorectal Cancer and Metastasis. Nat. Biotechnol. 35, 569–576. 10.1038/nbt.3836 28459449 PMC5462879

[B148] Roulis M. Kaklamanos A. Schernthanner M. Bielecki P. Zhao J. Kaffe E. (2020). Paracrine Orchestration of Intestinal Tumorigenesis by a Mesenchymal Niche. Nature 580, 524–529. 10.1038/s41586-020-2166-3 32322056 PMC7490650

[B149] Sachs N. de Ligt J. Kopper O. Gogola E. Bounova G. Weeber F. (2018). A Living Biobank of Breast Cancer Organoids Captures Disease Heterogeneity. Cell 172, 373–386. e310. 10.1016/j.cell.2017.11.010 29224780

[B150] Sachs N. Papaspyropoulos A. Zomer‐van Ommen D. D. Heo I. Böttinger L. Klay D. (2019). Long‐term Expanding Human Airway Organoids for Disease Modeling. Embo j 38, e100300. 10.15252/embj.2018100300 30643021 PMC6376275

[B151] Saini A. (2016). Cystic Fibrosis Patients Benefit from Mini Guts. Cell Stem Cell 19, 425–427. 10.1016/j.stem.2016.09.001

[B152] Sato T. Clevers H. (2013). Growing Self-Organizing Mini-Guts from a Single Intestinal Stem Cell: Mechanism and Applications. Science 340, 1190–1194. 10.1126/science.1234852 23744940

[B153] Sato T. Stange D. E. Ferrante M. Vries R. G. J. Van Es J. H. Van den Brink S. (2011). Long-term Expansion of Epithelial Organoids from Human colon, Adenoma, Adenocarcinoma, and barrett's Epithelium. Gastroenterology 141, 1762–1772. 10.1053/j.gastro.2011.07.050 21889923

[B154] Sato T. Vries R. G. Snippert H. J. van de Wetering M. Barker N. Stange D. E. (2009). Single Lgr5 Stem Cells Build Crypt-Villus Structures *In Vitro* without a Mesenchymal Niche. Nature 459, 262–265. 10.1038/nature07935 19329995

[B155] Schatoff E. M. Goswami S. Zafra M. P. Foronda M. Shusterman M. Leach B. I. (2019). Distinct Colorectal Cancer-Associated Apc Mutations Dictate Response to Tankyrase Inhibition. Cancer Discov. 9, 1358–1371. 10.1158/2159-8290.cd-19-0289 31337618 PMC6774804

[B156] Schnalzger T. E. Groot M. H. Zhang C. Mosa M. H. Michels B. E. Röder J. (2019). 3D Model for CAR ‐mediated Cytotoxicity Using Patient‐derived Colorectal Cancer Organoids. Embo j 38, e100928. 10.15252/embj.2018100928 31036555 PMC6576164

[B157] Schultz M. B. Vera D. Sinclair D. A. (2020). Can Artificial Intelligence Identify Effective COVID ‐19 Therapies? EMBO Mol. Med. 12, e12817. 10.15252/emmm.202012817 32569446 PMC7361072

[B158] Schuster B. Junkin M. Kashaf S. S. Romero-Calvo I. Kirby K. Matthews J. (2020). Automated Microfluidic Platform for Dynamic and Combinatorial Drug Screening of Tumor Organoids. Nat. Commun. 11, 5271. 10.1038/s41467-020-19058-4 33077832 PMC7573629

[B159] Shi R. Radulovich N. Ng C. Liu N. Notsuda H. Cabanero M. (2020a). Organoid Cultures as Preclinical Models of Non-small Cell Lung Cancer. Clin. Cancer Res. 26, 1162–1174. 10.1158/1078-0432.ccr-19-1376 31694835

[B160] Shi Y. Sun L. Wang M. Liu J. Zhong S. Li R. (2020b). Vascularized Human Cortical Organoids (Vorganoids) Model Cortical Development *In Vivo* . Plos Biol. 18, e3000705. 10.1371/journal.pbio.3000705 32401820 PMC7250475

[B161] Shu Q. Li W. Li H. Sun G. (2014). Vasostatin Inhibits Vegf-Induced Endothelial Cell Proliferation, Tube Formation and Induces Cell Apoptosis under Oxygen Deprivation. Ijms 15, 6019–6030. 10.3390/ijms15046019 24722573 PMC4013612

[B162] Siegel R. L. Fedewa S. A. Anderson W. F. Miller K. D. Ma J. Rosenberg P. S. (2017). Colorectal Cancer Incidence Patterns in the united states, 1974-2013. J. Natl. Cancer Inst. 109, djw322. 10.1093/jnci/djw322 PMC6059239 28376186

[B163] Siegel R. L. Torre L. A. Soerjomataram I. Hayes R. B. Bray F. Weber T. K. (2019). Global Patterns and Trends in Colorectal Cancer Incidence in Young Adults. Gut 68, 2179–2185. 10.1136/gutjnl-2019-319511 31488504

[B164] Smit W. L. Spaan C. N. Johannes de Boer R. Ramesh P. Martins Garcia T. Meijer B. J. (2020). Driver Mutations of the Adenoma-Carcinoma Sequence Govern the Intestinal Epithelial Global Translational Capacity. Proc. Natl. Acad. Sci. USA 117, 25560–25570. 10.1073/pnas.1912772117 32989144 PMC7568276

[B165] Song X. Shen L. Tong J. Kuang C. Zeng S. Schoen R. E. (2020). Mcl-1 Inhibition Overcomes Intrinsic and Acquired Regorafenib Resistance in Colorectal Cancer. Theranostics 10, 8098–8110. 10.7150/thno.45363 32724460 PMC7381732

[B166] Szvicsek Z. Oszvald Á. Szabó L. Sándor G. O. Kelemen A. Soós A. Á. (2019). Extracellular Vesicle Release from Intestinal Organoids Is Modulated by Apc Mutation and Other Colorectal Cancer Progression Factors. Cel. Mol. Life Sci. 76, 2463–2476. 10.1007/s00018-019-03052-1 PMC6529386 31028424

[B167] Takasato M. Er P. X. Chiu H. S. Maier B. Baillie G. J. Ferguson C. (2015). Kidney Organoids from Human Ips Cells Contain Multiple Lineages and Model Human Nephrogenesis. Nature 526, 564–568. 10.1038/nature15695 26444236

[B168] Takeda H. Kataoka S. Nakayama M. Ali M. A. E. Oshima H. Yamamoto D. (2019). Crispr-cas9-mediated Gene Knockout in Intestinal Tumor Organoids Provides Functional Validation for Colorectal Cancer Driver Genes. Proc. Natl. Acad. Sci. USA 116, 15635–15644. 10.1073/pnas.1904714116 31300537 PMC6681705

[B169] Takeishi K. Collin de l’Hortet A. Wang Y. Handa K. Guzman-Lepe J. Matsubara K. (2020). Assembly and Function of a Bioengineered Human Liver for Transplantation Generated Solely from Induced Pluripotent Stem Cells. Cel Rep. 31, 107711. 10.1016/j.celrep.2020.107711 PMC7734598 32492423

[B170] Tilg H. Adolph T. E. Gerner R. R. Moschen A. R. (2018). The Intestinal Microbiota in Colorectal Cancer. Cancer Cell 33, 954–964. 10.1016/j.ccell.2018.03.004 29657127

[B171] Tiriac H. Belleau P. Engle D. D. Plenker D. Deschênes A. Somerville T. D. D. (2018). Organoid Profiling Identifies Common Responders to Chemotherapy in Pancreatic Cancer. Cancer Discov. 8, 1112–1129. 10.1158/2159-8290.cd-18-0349 29853643 PMC6125219

[B172] Toden S. Ravindranathan P. Gu J. Cardenas J. Yuchang M. Goel A. (2018). Oligomeric Proanthocyanidins (Opcs) Target Cancer Stem-like Cells and Suppress Tumor Organoid Formation in Colorectal Cancer. Sci. Rep. 8, 3335. 10.1038/s41598-018-21478-8 29463813 PMC5820273

[B173] Touchefeu Y. Montassier E. Nieman K. Gastinne T. Potel G. Bruley des Varannes S. (2014). Systematic Review: The Role of the Gut Microbiota in Chemotherapy- or Radiation-Induced Gastrointestinal Mucositis - Current Evidence and Potential Clinical Applications. Aliment. Pharmacol. Ther. 40, a–n. 10.1111/apt.12878 25040088

[B174] Tran T. Q. Hanse E. A. Habowski A. N. Li H. Ishak Gabra M. B. Yang Y. (2020). α-Ketoglutarate Attenuates Wnt Signaling and Drives Differentiation in Colorectal Cancer. Nat. Cancer 1, 345–358. 10.1038/s43018-020-0035-5 32832918 PMC7442208

[B175] Tuveson D. Clevers H. (2019). Cancer Modeling Meets Human Organoid Technology. Science 364, 952–955. 10.1126/science.aaw6985 31171691

[B176] Tüysüz N. van Bloois L. van den Brink S. Begthel H. Verstegen M. M. A. Cruz L. J. (2017). Lipid-mediated Wnt Protein Stabilization Enables Serum-free Culture of Human Organ Stem Cells. Nat. Commun. 8, 14578. 10.1038/ncomms14578 28262686 PMC5343445

[B177] Ubink I. Bolhaqueiro A. C. F. Elias S. G. Raats D. A. E. Constantinides A. Peters N. A. (2019). Organoids from Colorectal Peritoneal Metastases as a Platform for Improving Hyperthermic Intraperitoneal Chemotherapy. Br. J. Surg. 106, 1404–1414. 10.1002/bjs.11206 31197820 PMC6771632

[B178] van de Wetering M. Francies H. E. Francis J. M. Bounova G. Iorio F. Pronk A. (2015). Prospective Derivation of a Living Organoid Biobank of Colorectal Cancer Patients. Cell 161, 933–945. 10.1016/j.cell.2015.03.053 25957691 PMC6428276

[B179] Verginelli F. Perconti S. Vespa S. Schiavi F. Prasad S. C. Lanuti P. (2018). Paragangliomas Arise through an Autonomous Vasculo-Angio-Neurogenic Program Inhibited by Imatinib. Acta Neuropathol. 135, 779–798. 10.1007/s00401-017-1799-2 29305721 PMC5904229

[B180] Vlachogiannis G. Hedayat S. Vatsiou A. Jamin Y. Fernández-Mateos J. Khan K. (2018). Patient-derived Organoids Model Treatment Response of Metastatic Gastrointestinal Cancers. Science 359, 920–926. 10.1126/science.aao2774 29472484 PMC6112415

[B181] Wang J. Huang F. Jiang C. Chi P. (2020). Silencing Signal Transducer and Activator of Transcription 3 (Stat3) and Use of Anti-programmed Cell Death-Ligand 1 (Pd-l1) Antibody Induces Immune Response and Anti-tumor Activity. Med. Sci. Monit. 26, e915854. 10.12659/msm.915854 32343679 PMC7201895

[B182] Wang R. Xiang W. Xu Y. Han L. Li Q. Dai W. (2020). Enhanced Glutamine Utilization Mediated by Slc1a5 and Gpt2 Is an Essential Metabolic Feature of Colorectal Signet Ring Cell Carcinoma with Therapeutic Potential. Ann. Transl Med. 8, 302. 10.21037/atm.2020.03.31 32355746 PMC7186745

[B183] Wang T. Pan W. Zheng H. Zheng H. Wang Z. Li J. J. (2021). Accuracy of Using a Patient-Derived Tumor Organoid Culture Model to Predict the Response to Chemotherapy Regimens in Stage IV Colorectal Cancer. Dis. Colon Rectum 64, 833–850. 10.1097/dcr.0000000000001971 33709991

[B184] Wang Y. Song W. Wang J. Wang T. Xiong X. Qi Z. (2020). Single-cell Transcriptome Analysis Reveals Differential Nutrient Absorption Functions in Human Intestine. J. Exp. Med. 217. 10.1084/jem.20191130 PMC7041720 31753849

[B185] Weeber F. van de Wetering M. Hoogstraat M. Dijkstra K. K. Krijgsman O. Kuilman T. (2015). Preserved Genetic Diversity in Organoids Cultured from Biopsies of Human Colorectal Cancer Metastases. Proc. Natl. Acad. Sci. USA 112, 13308–13311. 10.1073/pnas.1516689112 26460009 PMC4629330

[B186] Wen Y.-A. Xing X. Harris J. W. Zaytseva Y. Y. Mitov M. I. Napier D. L. (2017). Adipocytes Activate Mitochondrial Fatty Acid Oxidation and Autophagy to Promote Tumor Growth in colon Cancer. Cell Death Dis 8, e2593. 10.1038/cddis.2017.21 28151470 PMC5386470

[B187] Wong A. P. Bear C. E. Chin S. Pasceri P. Thompson T. O. Huan L.-J. (2012). Directed Differentiation of Human Pluripotent Stem Cells into Mature Airway Epithelia Expressing Functional Cftr Protein. Nat. Biotechnol. 30, 876–882. 10.1038/nbt.2328 22922672 PMC3994104

[B188] Workman M. J. Mahe M. M. Trisno S. Poling H. M. Watson C. L. Sundaram N. (2017). Engineered Human Pluripotent-Stem-Cell-Derived Intestinal Tissues with a Functional Enteric Nervous System. Nat. Med. 23, 49–59. 10.1038/nm.4233 27869805 PMC5562951

[B189] Wu S.-e. Hashimoto-Hill S. Woo V. Eshleman E. M. Whitt J. Engleman L. (2020). Microbiota-derived Metabolite Promotes Hdac3 Activity in the Gut. Nature 586, 108–112. 10.1038/s41586-020-2604-2 32731255 PMC7529926

[B190] Xie H. Zhang W. Zhang M. Akhtar T. Li Y. Yi W. (2020). Chromatin Accessibility Analysis Reveals Regulatory Dynamics of Developing Human Retina and Hipsc-Derived Retinal Organoids. Sci. Adv. 6, eaay5247. 10.1126/sciadv.aay5247 32083182 PMC7007246

[B191] Xu H. Jiao Y. Qin S. Zhao W. Chu Q. Wu K. (2018a). Organoid Technology in Disease Modelling, Drug Development, Personalized Treatment and Regeneration Medicine. Exp. Hematol. Oncol. 7, 30. 10.1186/s40164-018-0122-9 30534474 PMC6282260

[B192] Xu H. Lyu X. Yi M. Zhao W. Song Y. Wu K. (2018b). Organoid Technology and Applications in Cancer Research. J. Hematol. Oncol. 11, 116. 10.1186/s13045-018-0662-9 30219074 PMC6139148

[B193] Xu M. Lee E. M. Wen Z. Cheng Y. Huang W.-K. Qian X. (2016). Identification of Small-Molecule Inhibitors of Zika Virus Infection and Induced Neural Cell Death via a Drug Repurposing Screen. Nat. Med. 22, 1101–1107. 10.1038/nm.4184 27571349 PMC5386783

[B194] Xu Z. Liu T. Zhou Q. Chen J. Yuan J. Yang Z. (2019). Roles of Chinese Medicine and Gut Microbiota in Chronic Constipation. Evidence-Based Complement. Altern. Med. 2019, 1–11. 10.1155/2019/9372563 PMC6556327 31239866

[B195] Xuefeng X. Hou M.-X. Yang Z.-W. Agudamu A. Wang F. Su X.-L. (2020). Epithelial-mesenchymal Transition and Metastasis of colon Cancer Cells Induced by the Fak Pathway in Cancer-Associated Fibroblasts. J. Int. Med. Res. 48, 030006052093124. 10.1177/0300060520931242 PMC7323289 32588696

[B196] Yan H. H. N. Siu H. C. Law S. Ho S. L. Yue S. S. K. Tsui W. Y. (2018). A Comprehensive Human Gastric Cancer Organoid Biobank Captures Tumor Subtype Heterogeneity and Enables Therapeutic Screening. Cell Stem Cell 23, 882–897. e811. 10.1016/j.stem.2018.09.016 30344100

[B197] Yang T. Li X. Montazeri Z. Little J. Farrington S. M. Ioannidis J. P. A. (2019). Gene-environment Interactions and Colorectal Cancer Risk: An Umbrella Review of Systematic Reviews and Meta‐analyses of Observational Studies. Int. J. Cancer 145, 2315–2329. 10.1002/ijc.32057 30536881 PMC6767750

[B198] Yao Y. Xu X. Yang L. Zhu J. Wan J. Shen L. (2020). Patient-derived Organoids Predict Chemoradiation Responses of Locally Advanced Rectal Cancer. Cell Stem Cell 26, 17–26. e16. 10.1016/j.stem.2019.10.010 31761724

[B199] Yuan Y. Jiang Y.-C. Sun C.-K. Chen Q.-M. (2016). Role of the Tumor Microenvironment in Tumor Progression and the Clinical Applications (Review). Oncol. Rep. 35, 2499–2515. 10.3892/or.2016.4660 26986034

[B200] Zhang J. W. Du P. Chen D. W. Cui L. Ying C. M. (2010). Effect of Viable Bifidobacterium Supplement on the Immune Status and Inflammatory Response in Patients Undergoing Resection for Colorectal Cancer. Zhonghua Wei Chang Wai Ke Za Zhi 13, 40–43. 20099160

[B201] Zhao H. Yan C. Hu Y. Mu L. Liu S. Huang K. (2020). Differentiated Cancer Cell-Originated Lactate Promotes the Self-Renewal of Cancer Stem Cells in Patient-Derived Colorectal Cancer Organoids. Cancer Lett. 493, 236–244. 10.1016/j.canlet.2020.08.044 32898601

[B202] Zheng L. Wang B. Sun Y. Dai B. Fu Y. Zhang Y. (2021). An Oxygen-Concentration-Controllable Multiorgan Microfluidic Platform for Studying Hypoxia-Induced Lung Cancer-Liver Metastasis and Screening Drugs. ACS Sens. 6, 823–832. 10.1021/acssensors.0c01846 33657793

[B203] Zhou J. Su J. Fu X. Zheng L. Yin Z. (2017). Microfluidic Device for Primary Tumor Spheroid Isolation. Exp. Hematol. Oncol. 6, 22. 10.1186/s40164-017-0084-3 28794917 PMC5545869

